# Unraveling Core Functional Microbiota in Traditional Solid-State Fermentation by High-Throughput Amplicons and Metatranscriptomics Sequencing

**DOI:** 10.3389/fmicb.2017.01294

**Published:** 2017-07-14

**Authors:** Zhewei Song, Hai Du, Yan Zhang, Yan Xu

**Affiliations:** ^1^State Key Laboratory of Food Science and Technology, Key Laboratory of Industrial Biotechnology of Ministry of Education, Synergetic Innovation Center of Food Safety and Nutrition, School of Biotechnology, Jiangnan University Wuxi, China; ^2^State Key Laboratory of Microbial Metabolism, Joint International Research Laboratory of Metabolic and Developmental Sciences, School of Life Sciences and Biotechnology – Ministry of Education Key Laboratory of Systems Biomedicine, Shanghai Center for Systems Biomedicine, Shanghai Jiao Tong University Shanghai, China

**Keywords:** fermentation microbiota, core functional microbiota, solid-state fermentation, microbial succession, functional shift, high-throughput sequencing, amplicons, metatranscriptomics

## Abstract

Fermentation microbiota is specific microorganisms that generate different types of metabolites in many productions. In traditional solid-state fermentation, the structural composition and functional capacity of the core microbiota determine the quality and quantity of products. As a typical example of food fermentation, Chinese Maotai-flavor liquor production involves a complex of various microorganisms and a wide variety of metabolites. However, the microbial succession and functional shift of the core microbiota in this traditional food fermentation remain unclear. Here, high-throughput amplicons (16S rRNA gene amplicon sequencing and internal transcribed space amplicon sequencing) and metatranscriptomics sequencing technologies were combined to reveal the structure and function of the core microbiota in Chinese soy sauce aroma type liquor production. In addition, ultra-performance liquid chromatography and headspace-solid phase microextraction-gas chromatography-mass spectrometry were employed to provide qualitative and quantitative analysis of the major flavor metabolites. A total of 10 fungal and 11 bacterial genera were identified as the core microbiota. In addition, metatranscriptomic analysis revealed pyruvate metabolism in yeasts (genera *Pichia, Schizosaccharomyces, Saccharomyces*, and *Zygosaccharomyces*) and lactic acid bacteria (genus *Lactobacillus*) classified into two stages in the production of flavor components. Stage I involved high-level alcohol (ethanol) production, with the genus *Schizosaccharomyces* serving as the core functional microorganism. Stage II involved high-level acid (lactic acid and acetic acid) production, with the genus *Lactobacillus* serving as the core functional microorganism. The functional shift from the genus *Schizosaccharomyces* to the genus *Lactobacillus* drives flavor component conversion from alcohol (ethanol) to acid (lactic acid and acetic acid) in Chinese Maotai-flavor liquor production. Our findings provide insight into the effects of the core functional microbiota in soy sauce aroma type liquor production and the characteristics of the fermentation microbiota under different environmental conditions.

## Introduction

The microbiota in natural environments is complex and often includes thousands of genera from a diverse range of species (Astudillo-Garcia et al., 2017). In industrial fermentation, the microbiota depends on the conditions and nutrient availability (Sekwati-Monang et al., 2012). In natural environments or under artificially controlled conditions, fermentation microbiota have the potential to use different raw materials to produce a variety of metabolites (Xu and Ji, 2012; Lu et al., 2016; Wang et al., 2016). They are commonly characterized by the presence of specific microorganisms, orderly microbial succession and an unusual functional shift (Azwar et al., 2014; Di Cagno et al., 2014; Kong et al., 2014; Qi et al., 2014). As an example of a typical process involving fermentation microbiota, traditional solid-state fermentation (SSF) generates fermented foods that have a high nutritional value and confer health benefits due to the involvement of multiple microorganisms and biochemical reactions (Giraffa, 2004; Kong et al., 2014; Tamang et al., 2016).

In recent years, the concept of core microbiota has emerged (Astudillo-Garcia et al., 2017). The core microbiota play important roles in natural ecological systems, human health and industrial processes (Schmitt et al., 2012; Zhang et al., 2015; Kable et al., 2016). However, the core microbiota that influence the quantity and quality of fermented foods in traditional SSF are poorly understood (Rantsiou and Cocolin, 2006; Cocolin et al., 2013). Traditionally, species were isolated and identified from food fermentation processes by culture-dependent and -independent techniques (Papalexandratou et al., 2013; Chen et al., 2014; Ricciardi et al., 2015). Although these studies gained some insight into the microbial populations, our knowledge about the core structural microbiota in fermented foods remain limited (Wu et al., 2013). With the development of high-throughput sequencing techniques, researches have focused on the structure of the core microbiota in traditional SSF processes (Ercolini, 2013; Aldrete-Tapia et al., 2014). For example, in cheese production, 10 fungal genera and 14 bacterial genera (taxa > 1%) are considered the core microbiota by 16S rRNA gene and internal transcribed spacer (ITS) amplicon sequencing (Wolfe et al., 2014). Similarly, in soy sauce fermentation, 22 frequent genera have been reported to be candidates of the core microbiota (Sulaiman et al., 2014).

Although the above-mentioned studies illustrate the structure of the core microbiota in traditional fermented foods, the correlation between the microbiota and metabolites during different processes remains unclear (Papagianni, 2014). To address this, research has focused on the function of the core microbiota in traditional food fermentation processes (Garrote et al., 2015). Recently, the high-throughput meta-omics sequencing technique has been demonstrated to be informative in discovering microbial succession patterns and the function shift that are characteristic of the fermentation microbiota and are crucial for their function (Bokulich and Mills, 2012; Solieri et al., 2013). For example, in cheese production, metatranscriptomic sequencing demonstrated that some lactic acid bacteria (LAB) are considered the main functional contributors in the processes of proteolysis, lipolysis, and amino acid/lipid catabolism (De Filippis et al., 2016). Similarly, metagenomic sequencing revealed that in pickle production, the genera *Novosphingobium* and *Sphingomonas* are starter microorganisms, which are subsequently replaced by the genera *Leuconostoc, Lactobacillus*, and *Weissella*. In addition, metatranscriptomic sequencing analysis revealed that the genes related to carbohydrate transport, hydrolysis, and pyruvate metabolism are actively expressed and highly improve the metabolic capacity of lactic acid production (Jung et al., 2013).

Chinese soy sauce aroma type (Maotai-flavor) liquor production (Supplementary Figure S1) is a spontaneous and repeated batch process thought to originate from ancient fermented beverages (McGovern et al., 2004). In addition to the common characteristics of traditional SSF (Thomas et al., 2013), soy sauce aroma liquor production also displays some specific characteristic features influenced by endogenous factors (such as temperature, pH, moisture, ethanol, lactic acid, and acetic acid) in the pit fermentation process (**Table 1**). The fermentation mechanism required for Chinese soy sauce aroma liquor production therefore involves complex microbiota and metabolites (Xu et al., 2010). Moreover, the fermentation microbiotas use cereals (major sorghum and wheat) as raw materials to form a variety of flavor components through a series of biochemical reactions, including various carbon, nitrogen, and sulfur metabolites (Zhu et al., 2007; Xu et al., 2010; Wu et al., 2012). However, the functional correlation between the core microbiota and important metabolites remains to be established in soy sauce aroma liquor production. More specifically, microbial succession and the functional shift in the core microbiota have not been clarified to date. Therefore, in this study, we employed several high-throughput sequencing technologies (16S rRNA gene amplicon sequencing, ITS amplicon sequencing and metatranscriptomics sequencing) to explore the structure and function of core microorganisms in fermentation microbiota (Schoch et al., 2012; Fierer et al., 2013; Glass et al., 2015). In addition, we combined ultra-performance liquid chromatography (UPLC) and headspace-solid phase microextraction-gas chromatography-mass spectrometer (HS-SPME-GC-MS) to explore the fluctuations in major flavor components (Cordovez et al., 2015; Zothanpuia et al., 2017). Based on this information, we explored the correlations between the core microbiota and important metabolites.

**Table 1:** Correlation between microbiota and endogenous factors.

Sample		Temperature (°C)		pH		Moisture (%)		Ethanol (g/kg fermented grain)		Lactic acid (g/kg fermented grain)		Acetic acid (g/kg fermented grain)	
		Range	*P*^a^	Range	*P*^a^	Range	*P*^a^	Range	*P*^a^	Range	*P*^a^	Range	*P*^a^
Time	Day 5 (*n* = 3)	36.3–42.8	0.800	3.65–3.70	0.008	55.65–55.69	0.001	10.30–34.49	0.381	27.07–28.80	0.046	15.75–23.92	0.235
	Day 10 (*n* = 3)	38.2–41.7		3.64–3.69		55.62–55.65		17.60–49.99		26.94–29.23		15.74–18.24	
	Day 15 (*n* = 3)	32.7–41.2		3.64–3.70		55.44–55.51		26.87–55.91		24.28–30.47		19.33–24.13	
	Day 20 (*n* = 3)	30.5–40.7		3.61–3.66		54.71–57.73		28.02–49.25		26.71–32.72		16.85–21.08	
	Day 25 (*n* = 3)	29.8–40.5		3.61–3.64		55.69–55.64		34.55–50.74		27.74–30.30		16.30–18.17	
	Day 30 (*n* = 3)	28.7–39.8		3.55–3.59		54.68–54.72		24.16–50.24		30.85–43.00		19.39–32.25	

*^a^The P-value was corrected with different group of samples by ANOVA.*

## Materials and Methods

### Sample Collection

All samples were collected in Xishui County (28.14 N, 106.18 E), Guizhou Province, China. The fermentation process was carried out in four distinct phases: starter (Daqu) making, stacking fermentation, pit fermentation, and distillation. Samples were taken during the pit fermentation phase of liquor production (Supplementary Figure S1). Raw materials (15 tons) were mixed with sorghum and wheat in a pit (3 m × 2.5 m × 4 m). In addition, they were also selected from different locations (points A, B, and C) in the pit. Samples were collected at 5-day intervals in duplicate, one for genomic DNA extraction and total RNA extraction, the other for flavor component analysis. Samples from the three points (1, 2, and 3) were mixed in the same layer to form one sample to reduce the volatility of samples before extraction and analysis.

### DNA Extraction, 16S rRNA Gene Amplicon Sequencing, and ITS Amplicon Sequencing

All samples were treated with sterile phosphate-buffered saline (PBS, 0.1 mol/L), then centrifuged at 30 × *g* for 10 min. The supernatant was then centrifuged again at 10,000 rpm for 10 min. The precipitated cells were milled with liquid nitrogen and genomic DNA was extracted using sodium laurate buffer (sodium laurate 10 g/L, Tris-HCl 0.1 mol/L, NaCl 0.1 mol/L, ethylenediaminetetraacetic acid (EDTA) 0.02 mol/L) containing phenol:chloroform:isoamyl alcohol (25:24:1). Bacterial V3-V4 and the fungal ITS1 of the rRNA were amplified using forward primers (5′-GTACTCCTACGGGAGGCAGCA-3′, 5′-CTTGGTCATTTAGAGGAAGTAA-3′) and the reverse primer (5′-GTGGACTACHVGGGTWTCTAAT-3′, 5′-TGCGTTCTTCATCGATGC-3′), respectively. Amplicons were then sequenced using an Illumina MiSeq platform (Illumina, San Diego, CA, United States) at AuwiGene Technology Co., Ltd., Beijing, China.

### 16S rRNA Gene and ITS Amplicon Data Analyses

Raw sequences were processed using ‘quantitative insights into microbial ecology’ software (QIIME, version 1.8) (Caporaso et al., 2010). Briefly, sequences with ambiguous bases (‘N’) were removed, and reads were quality-filtered to remove bases with Q < 0.02 by Trimmomatic (version 0.32) (Bolger et al., 2014). Then, overlapping reads merged by Fastq-join (version 1.8) and primer sequences were removed and complete assembled reads were used for further analysis. The sequences were clustered into operational taxonomic units (OTUs) at 97% sequence similarity and chimeras were removed by USEARCH (version 8.1.1861) (Angly et al., 2016). In addition, OTUs of 16S rRNA gene amplicons were mapped to Silva (version 119) (Zhu et al., 2015) and OTUs of ITS amplicons were mapped to Unite (version 7.0) (Kõljalg et al., 2013). Fungal OTUs were manually corrected using the CBS database (Centraalbureau voor Schimmelcultures, Holland) (Hallin and Ussery, 2004) and bacterial OTUs were manually corrected by Ezbiocloud (The Jongsik Chun Lab. and Chunlab, Inc., South Korea) (Gulati et al., 2015). The amplicon databases have been submitted to NCBI SRA and are available under accession numbers SRR5298103 and SRR5298193.

### Total RNA Extraction and Metatranscriptomic Sequencing

The initial steps of total RNA extraction were the same as those for DNA extraction. After centrifugation, precipitated cells were milled with liquid nitrogen and total RNA was extracted with sodium laurate buffer (sodium laurate 10 g/L, Tris-HCl 0.1 mol/L, NaCl 0.1 mol/L, EDTA 0.02 mol/L) containing TRIzol (Sigma-Aldrich, St. Louis, MO, United States). Ribo-Zero^TM^ rRNA Removal Kits (Bacteria) and Ribo-Zero^TM^ Magnetic Gold Kit (Yeast) (Epicentre, San Diego, CA, United States) were used to remove rRNA from the total RNA. Metatranscriptomic libraries were constructed according to the NEBNext^®^ Ultra^TM^ RNA Library Prep Kit (Illumina, New England Biolabs), and were then sequenced on an Illumina Hiseq 2500 platform at the Biomarker Technologies Co., Ltd., Beijing, China (Ziesemer et al., 2015).

### Metatranscriptomic Data Analyses

Raw metatranscriptomic data were processed by removing the rRNA sequences and low quality reads (Q < 0.02). Trinity (version 2.1.1) (Haas et al., 2013) was employed for *de novo* transcriptomic assemblies. Using DIAMOND (version 0.8.34) (Buchfink et al., 2015) and inputting results into MEGAN (version 5.11.3) (Huson et al., 2007), we obtained microbial taxonomy information. Then, we compared high quality reads to the non-redundant protein database (Liu et al., 2013) and the KEGG database (Kanehisa et al., 2004) to obtain functional annotation information. Heatmap Illustrator (HemI, v.1.0) (Deng et al., 2014) was used to illustrate different gene expression patterns in the main metabolic pathways. SPSS (version 19.0, Web Atlas, Paris, France) and HemI were used to establish correlations generated by the metatranscriptomic database analysis (*P* < 0.05). Other statistical analyses and graphics were executed using Microsoft^®^ Excel and R software (version 3.3.2). The metatranscriptomic database has been submitted to NCBI SRA and is accessible under SRR5306242.

### Flavor Components Analyses

The flavor components in samples were analyzed by HS-SPME-GC-MS and UPLC. For HS-SPME-GC-MS analysis, samples were treated as follows: 4 g of each sample was selected and placed in 50 ml centrifuge tubes. Firstly, 40 ml of sterile distilled water and 0.4 g of CaCl_2_ were added to the sample and mixed. Secondly, tubes were placed in ice water for 30 min. Finally, the supernatant was collected by centrifuging at 8000 rpm for 10 min. The automatic headspace sampling system (Multi-Purpose Sample MPS 2 with a SPME adapter) (GERSTEL Inc., Baltimore, MD, United States) with a 50/30 μm DVB/CAR/PDMS fiber (Supelco Inc., Bellefonte, PA, United States) was used in SPME. Samples were preheated for 5 min and extracted for 45 min at 50°C. The Agilent 6890N GC coupled with an Agilent 5975 Mass Selective Detector was used in GC-MS. The GC-MS conditions were: a starting temperature of 50°C (held for 2 min), then increased to 230°C at a rate of 4°C/min and held at 230°C for 15 min. For UPLC analysis, we used the same method as previously described without 0.4 g CaCl_2_. The UPLC system consisted of an Acquity UPLC (Waters, Milford, MA, United States) coupled with an Acquity UPLC Tunable UV Detector (Waters). The column was an Acquity UPLC HSS T3 column (2.1 mm × 100 mm, 1.8 μm) and the eluent was NaH_2_PO_4_. The mass spectra of unknown flavor components were compared with those in the Wiley 275.L (Agilent) database.

## Results

### Major Alcohols and Acids in the Flavor Components of Soy Sauce Aroma Liquor Production

**FIGURE 1:**
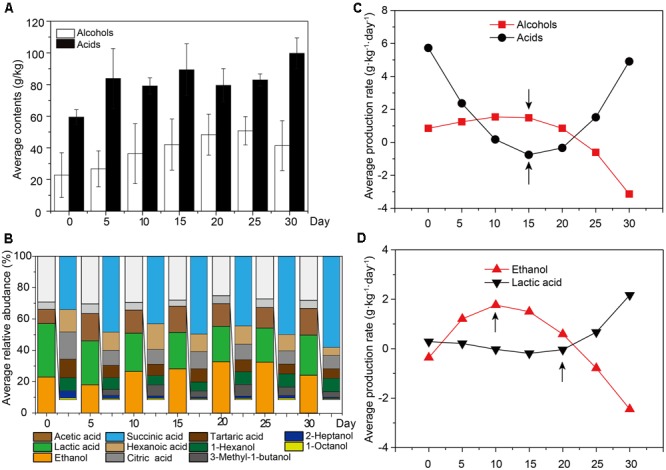
The average content of flavor components across all the samples. Samples were sorted based on the fermentation time. **(A)** Average content of alcohols and acids across all the samples (*n* = 18, each bar *n* = 3). **(B)** Average relative abundance of major alcohols and acids across all the samples (*n* = 18, each bar *n* = 3). **(C)** The production rate of major alcohols and acidsacross all the samples (*n* = 18). **(D)** The production rate of ethanol and lactic acidacross all the samples (*n* = 18).

To examine the flavor components of liquor production, important non-volatile and volatile components were qualified and quantified by UPLC and HS-SPME-GC-MS across samples. A total of 61 major components were identified, including 10 alcohols (mainly ethanol, 1-hexanol and 3-methyl-1-butanol), 12 acids (mainly lactic acid, acetic acid and succinic acid) and 28 esters (mainly ethyl acetate, isobutyl hexanoate, and diethyl succinate) (**Figure 1A** and Supplementary Figure S2). It was apparent that ethanol was the most abundant metabolite (18.87 ± 14.98 – 43.43 ± 8.21 g/kg fermented grain), followed by lactic acid (28.05 ± 4.75 – 36.20 ± 6.20 g/kg fermented grain) and acetic acid (7.40 ± 2.05 – 23.98 ± 7.18 g/kg fermented grain) (Supplementary Figure S2). The relative abundance of alcohols increased from days 0 to 15, with the average ranging from 24.82 to 32.35%. Among the alcohols, the relative abundance of ethanol increased from days 5 to 15, with the average ranging from 18.02 to 28.18% (**Figure 1B**). By contrast, the relative abundance of acids increased from days 15 to 30, ranging from 62.70 to 70.34% (**Figure 1B**). Among the acids, the relative abundance of lactic acid and acetic acid increased from days 15 to 30, with the average ranging between 34.81 and 42.62% (**Figure 1B**). The production rate of the major flavor components is shown in Supplementary Figure S3. The production rate of alcohols decreased from days 0 to 10, with the average ranging from 0.85 to 1.54 for alcohols (**Figure 1C**), and more specifically, from -0.36 to 1.77 for ethanol (**Figure 1D**). By contrast, the production rate of acids increased from days 20 to 30, with the average ranging from -0.34 to 4.91 for acids generally (**Figure 1C**) and -0.01 to 2.17 for lactic acid and acetic acid specifically (**Figure 1D**).

### Endogenous Factors Distinctly Reflected Microbial Succession in the Fermentation Microbiota

To understand the composition of the microbiota, OTU reads were selected to depict the bacterial and fungal microbiota in liquor production (Stegen et al., 2016). A total of 712,610 high quality reads were generated from bacterial 16S rDNA V3–V4 sequences in 18 samples, ranging from 15,272 to 106,976 reads. We also obtained 912,794 high quality reads from fugal ITS1 sequences in 18 samples, ranging from 27,240 to 77,221 reads. In addition, 16S rRNA gene amplicons sequences clustered into 522 OTUs and ITS amplicons sequences clustered into 552 OTUs at a 97% similarity level. Bacterial sequence numbers were normalized to 12,426 reads and fungal sequence numbers were normalized to 27,470 reads for describing the composition characteristics (Supplementary Tables S1, S2). The *Good’s coverage* estimator (Long et al., 2016a) revealed that 99.55–99.74% of bacterial and fungal OTUs were obtained in all samples, suggesting that major bacterial and fungal OTUs had been captured. The bacterial *Chao1* richness estimator (Long et al., 2016b) decreased with a range from 296.22 ± 23.22 to 160.07 ± 3.03 at different sampling times, whereas the fungal *Chao1* richness estimator increased with a range from 153.91 ± 37.31 to 262.16 ± 4.60 (Supplementary Figure S3 and Tables S1, S2). In addition, the bacterial *Shannon* diversity index (Chen et al., 2015) decreased with a range from 4.54 ± 0.69 to 0.26 ± 0.04 at different sampling times, whereas the fungal *Shannon* diversity index increased with a range between 1.77 ± 0.12 and 3.09 ± 0.55 (Supplementary Figure S3 and Tables S1, S2).

**FIGURE 2:**
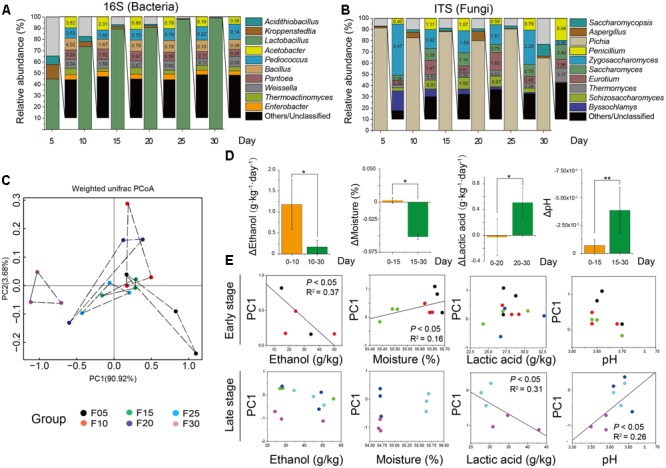
Microbiota and major flavor components analysis across all the samples. Average bacterial **(A)** and fungal **(B)** distribution at the genus-level in microbiota based on 16S rRNA gene and ITS amplicons (*n* = 18, each bar *n* = 3). **(C)** Amplicons analysis represented the similarities of microbial compositions based on principal component analysis (PCA). **(D)** Ethanol, lactic acid, and acetic acid production had different stages. Bars represented mean (±SE). Asterisk indicates significant differences by Mann–Whitney *U* test (*P* < 0.05). **(E)** Plots of PC1 versus four factors showed that ethanol and lactic acid had significant correlation with microbiota in different stages by Spearman correlation coefficient (*P* < 0.05).

Sequence similarity thresholds (80 to 100%) were used to define the OTUs. The range of similarity cut-offs, 80, 90, 97, and 99%, nominally estimated phylum, class, genus, and species, respectively (Whiteson et al., 2014). *Firmicutes* and *Ascomycota* were dominant at the phylum level, comprising about 90% of bacteria and 95% of fungi (**Figure 2A**). The largest groups of bacterial genera were *Lactobacillus, Kroppenstedtia, Acidithiobacillus, Acetobacter*, and *Pediococcus*, whereas the largest groups of fungal genera were *Pichia, Aspergillus, Saccharomycopsis, Penicillium*, and *Zygosaccharomyces* (**Figure 2B**). The genus *Lactobacillus* (on average accounting for 44.86 to 98.83% of bacteria) was the most abundant bacterial genus. The genus *Pichia* (on average accounting for 91.34 to 64.59% of fungi) was the most abundant fungal genus in each sample. The overall abundances of bacterial genera showed that the average relative abundance of the genus *Lactobacillus* increased but the abundance of the genera *Kroppenstedtia* (12.92–0.12%) and *Acidithiobacillus* (7.55–0.12%) decreased at different sampling times. Simultaneously, the overall relative abundances of fungal genera also changed. The relative abundance of the genus *Pichia* decreased but the genera *Aspergillus* (0.50–8.42%) and *Saccharomycopsis* (0.35–10.61%) increased at different sampling times.

Based on 16S rRNA and ITS gene amplicons, Unifrac distance-based weighted principal coordinates analysis was conducted to evaluate similarities and differences in the microbiota (prokaryotic and eukaryotic microbiota) among the different samples (**Figure 2C**). Although there was a slight difference in the microbiota among samples, similar clustering patterns were observed. Samples from days 15, 20, and 25 formed clusters. Samples from days 15 and 20 were distributed similarly to samples from day 25, whereas samples from days 5 and 30 were dispersed within other samples. The differences between samples on days 5 and 30 were larger than the differences between other samples. To demonstrate the major factors associated with microbial succession at the different stages of liquor production, multiple endogenous factors (temperature, pH, moisture, ethanol, lactic acid, and acetic acid) were selected and analyzed to reveal their connection with the microbiota (**Table 1**). We found that the temperature and acetic acid content of samples showed no significant difference (*P* > 0.05) as determined by a Mann–Whitney *U* test (Supplementary Figure S4). However, the ethanol content of samples from days 0 to 10 differed significantly (*P* < 0.05) compared with samples from days 10 to 30. The moisture levels of samples also differed significantly for days 0 to 15 (*P* < 0.05) compared with samples from days 15 to 30 (**Figure 2D**) as determined by a Mann–Whitney *U* test. The Mann–Whitney *U* test also revealed a significant difference in the lactic acid content of samples from days 0 to 20 (*P* < 0.05) compared with samples from days 20 to 30 and pH of samples from days 0 to 15 was significantly different (*P* < 0.01) compared with that of samples from days 15 to 30 (**Figure 2D**). Ethanol and moisture were the best indicators in the early stage of liquor production, with principal coordinate 1 (PC1) being significantly associated with the ethanol content of samples from days 5 to 10 (*R*^2^ = 0.45, *P* < 0.05) and the moisture content of samples from days 25 to 30 (*R*^2^ = 0.56, *P* < 0.05) (**Figure 2D**). Lactic acid and pH were the best indicators in the late stage of liquor production, with principal coordinate 1 (PC1) being significantly correlated with the lactic acid content of samples from days 25 to 30 (*R*^2^ = 0.61, *P* < 0.05) and the pH of samples from days 25 to 30 (*R*^2^ = 0.52, *P* < 0.01) (**Figure 2E**).

### Core Microbiota Showed a Significant Correlation with Major Endogenous Factors at Different Stages of Production

**FIGURE 3:**
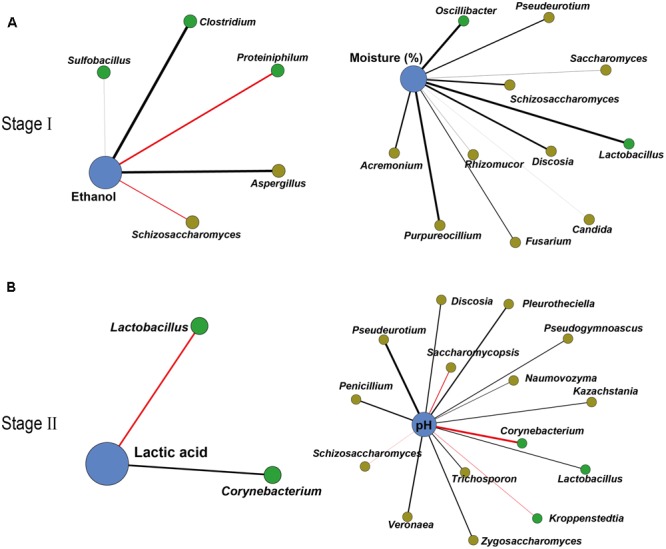
Correlation network between microbiota and endogenous factors. Spearman correlation coefficient depicted significant negative and positive correlations (*P* < 0.05) between microbiota and endogenous factors in Stage I **(A)** and Stage II **(B)** (relative abundance > 0.01%). Edge thickness represented the value. Edge color represented the positive (red) or negative (black) correlation. Edge length and point sizes had no meaning for the present study.

To further illustrate the correlation between the core microbiota and major endogenous factors, amplicons and the major endogenous factor database were combined to explore positive and negative correlations between the microbiota and major endogenous factors using Gephi (version 0.9.1) (**Figure 3** and Supplementary Tables S3, S4). In total, 33 pairs of significant and robust relationships (edges) were identified from 33 genera (nodes) (*P* < 0.05). Gephi network analysis (Jacomy et al., 2009) detected 7 positive and 26 negative correlations using Spearman’s correlation coefficient (Borkowf, 2002), revealing that the genera *Sulfobacillus, Clostridium*, and *Aspergillus* negatively correlated with ethanol (*P* < 0.05) in the early stage of liquor production (Stage I). The genera *Proteiniphilum* and *Schizosaccharomyces* showed a positive correlation with ethanol (*P* < 0.05) in Stage I. Bacterial genera (*Lactobacillus* and *Oscillibacter*) and fungal genera (*Saccharomyces, Schizosaccharomyces, Purpureocillium, Pseudeurotium, Candida, Fusarium, Rhizomucor, Discosia*, and *Acremonium*) displayed a negative correlation with moisture (*P* < 0.05) in Stage I. By contrast, in the late stage of liquor production (Stage II), the genus *Corynebacterium* showed a negative correlation with lactic acid, whereas the genus *Lactobacillus* showed a positive correlation with lactic acid (*P* < 0.05). The bacterial genus *Lactobacillus* and various fungal genera (*Penicillium, Zygosaccharomyces, Pleurotheciella, Naumovozyma, Kazachstania, Trichosporon, Pseudeurotium, Veronaea, Discosia*, and *Pseudogymnoascus*) displayed a negative correlation with pH (*P* < 0.05) in Stage II. The bacterial genera (*Kroppenstedtia* and *Corynebacterium*) and the fungal genera (*Saccharomycopsis* and *Schizosaccharomyces*) showed a positive correlation with pH (*P* < 0.05) in Stage II.

### Variations in Pyruvate Metabolism Reflected a Functional Shift in the Fermentation Microbiota

To explore microbial function in Chinese soy sauce aroma liquor production, the KEGG database was used to explore the overall metabolic capability of microbiota *in situ* by HemI (Supplementary Figure S5). Among 64,349 unigenes (i.e., transcripts that originate from the same transcription locus) related to major KEGG metabolic pathways, 9,003 unigenes (13.99%) were attributed to metabolic pathways involved in maintaining the basic survival of microorganisms (ribosomes, cell cycle, and RNA transport), whereas the others (86.01%) were attributed to different types of flavor components. These unigenes were attributed to carbon metabolic pathways (27,788, 43.18%), nitrogen metabolic pathways (20,667, 32.12%) and other metabolic pathways (6,891, 10.71%). The most abundant type of metabolism in carbon metabolic pathways was glycolysis/gluconeogenesis (1,777, 2.76%), followed by pyruvate metabolism (1,480, 2.30%) and the tricarboxylic acid cycle (TCA cycle) (1,433, 2.23%).

**FIGURE 4:**
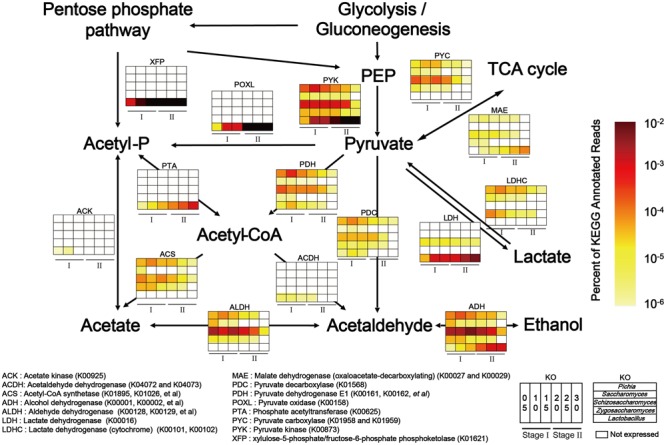
Relative abundance of KEGG expression genes in pyruvate metabolism related to genera *Pichia, Schizosaccharomyces, Saccharomyces, Zygosaccharomyces*, and *Lactobacillus* in different stages (Stage I: samples from days 5 to 15 and Stage II: samples from days 20 to 30 (*n* = 6). Color depth represented the proportion of total reads mapping to KEGG metabolic pathway in all KEGG annotated reads.

To explain the correlations between core microorganisms and major endogenous factors in the microbiota, the KEGG and non-redundant protein databases were combined to illustrate pyruvate metabolism related to ethanol, lactic acid and acetic acid production. In our study, the genera *Pichia, Saccharomyces, Schizosaccharomyces, Zygosaccharomyces*, and *Lactobacillus* were the dominant functional contributors to the fermentation microbiota (Supplementary Figure S6). Functional genes related to the genera *Pichia, Saccharomyces, Schizosaccharomyces, Zygosaccharomyces*, and *Lactobacillus* were expressed at high levels in pyruvate metabolism (**Figure 4**). Lactate dehydrogenase (cytochrome) (LDHC, K00101, and K00102) and pyruvate dehydrogenase E1 component (PDH, K00161, K00162 and K00163) peaked in expression in the genus *Pichia* during pyruvate metabolism. Pyruvate kinase (PYK, K00873), aldehyde dehydrogenase (e.g., ADH, K00128, K00129) and alcohol dehydrogenase (e.g., ADH, K00001, and K00002) showed the highest levels of gene expression in the genera *Schizosaccharomyces* and *Lactobacillus* during carbohydrate metabolism. Although the genera *Pichia, Schizosaccharomyces, Saccharomyces, Zygosaccharomyces*, and *Lactobacillus* shared the same pathway, high level gene expression occurred in different samples. Moreover, to maintain the high-level expression of pyruvate metabolism, PYK became the predominant KEGG gene in the genera *Pichia, Schizosaccharomyces, Saccharomyces, Zygosaccharomyces*, and *Lactobacillus*. Consistent with the need to use lactic acid and ensure the conversion of pyruvate to acetaldehyde and acetyl-CoA, our results showed that LDH, PDH and pyruvate decarboxylase (PDC, K01568) became the major KEGG genes in the genus *Pichia*. KEGG expression genes related to the genus *Schizosaccharomyces* showed high metabolic capacity via PDH, acetyl-CoA synthetase (ACS, K01895, K01067, etc.), ALDH and ADH, which converted pyruvate to acetaldehyde through acetyl-CoA and acetate, and then converted acetaldehyde to ethanol in the fermentation process. In addition, KEGG genes related to genus *Lactobacillus* showed high metabolic capacity via LDH, xylulose-5-phosphate/fructose-6-phosphate phosphoketolase (XFP, K01621), phosphate acetyltransferase (PTA, K00625) and ADH in liquor production.

**FIGURE 5:**
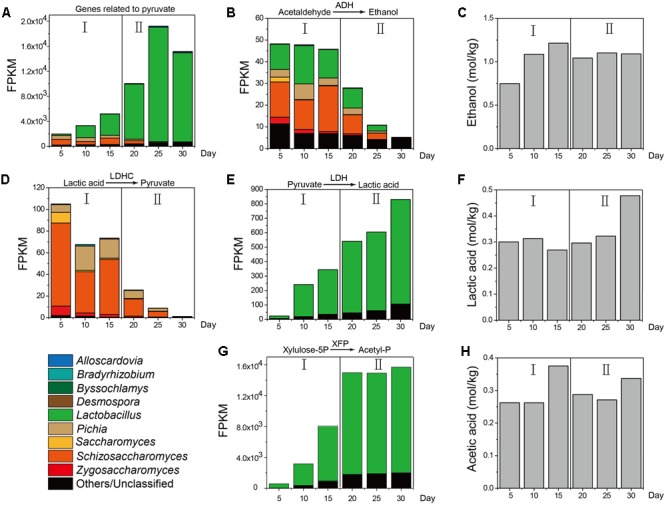
KEGG expression genes in pyruvate metabolism associated with the measured content of ethanol, lactic acid, and acetic acid in different stages (*n* = 6). **(A)** FPKM (Fragments per Kilobase of transcript per Million mapped reads) of KEGG expression genes related to pyruvate. **(B)** FPKM of KEGG expression genes related to metabolic pathway from acetaldehyde to ethanol. **(C)** Ethanol content from Stage I to Stage II. **(D)** FPKM of KEGG expression genes related to metabolic pathway from lactic acid to pyruvate. **(E)** FPKM of KEGG expression genes from pyruvate to lactic acid. **(F)** Lactic acid content from Stage I to Stage II. **(G)** FPKM of KEGG expression genes from pyruvate to acetyl-phosphate. **(H)** Acetic acid content from Stage I to Stage II.

Furthermore, as a major metabolic pathway, pyruvate metabolism became increasingly active during the temporal course, as determined by the FPKM (Fragments per Kilobase of transcript per Million mapped reads) method (Guo et al., 2013) (**Figure 5**). ADH in molds (genera *Aspergillus* and *Penicillium*), yeasts (genera *Pichia, Schizosaccharomyces, Saccharomyces*, and *Zygosaccharomyces*) and LAB (genus *Lactobacillus*) displayed different metabolic capacities to form ethanol in different samples, which explained the variation in ethanol content between samples during production (**Figures 5B,C**). LDHC could convert lactic acid to pyruvate and its gene expression levels were obviously downregulated from 105.05 to 1.20 in mold (genus *Byssochlamys*) and yeasts (genera *Pichia, Zygosaccharomyces*, and *Saccharomyces*) from days 10 to 30 (**Figure 5D**). By contrast, LDH could convert pyruvate to lactic acid and its gene expression levels were upregulated from 23.86 to 830.18 in yeast (genus *Schizosaccharomyces*) and LAB (genus *Lactobacillus*) from days 5 to 30 (**Figure 5E**). The lactic acid content (**Figure 5F**) correlated with the functional shift in lactic acid production. PYC and malate dehydrogenase (oxaloacetate-decarboxylating) (e.g., MAE, K00027, K00028) could convert pyruvate in the TCA cycle and their gene expression levels fluctuated in different samples. XFP could convert glucose to acetyl phosphate without pyruvate metabolism and expression of this gene was upregulated from 556.43 to 13,668.3 in genus *Lactobacillus* from days 5 to 30 (**Figure 5G**). Conversely, the acetic acid content fluctuated over time (**Figure 5H**).

## Discussion

In Chinese soy sauce aroma liquor production, the core microorganisms play critical roles in the characteristics of the flavor metabolites. Compared with previous studies (Wu et al., 2013; Chen et al., 2014), we found that 11 bacterial genera and 10 fungal genera were the core microbiota in the microbial succession of liquor production, which affected the production of flavor components (**Figures 2A,B**). Compared with previous studies (Wang X. et al., 2014), we found that samples with a high alcohol content differed from those with a high acid content in the production process. The alcohol content increased quickly in samples from days 0 to 10, whereas the acid content increased rapidly from days 20 to 30 (**Figure 1**). In addition, the production rate of alcohol content decreased quickly in samples from days 0 to 15, whereas the acid content increased quickly at samples from days 15 to 30 (**Figures 1C,D**). There was a significant difference (*P* < 0.05) between the ethanol and lactic acid contents at different stages of liquor production (**Figure 2D** and Supplementary Figure S4). Ethanol and moisture level significantly correlated with the fermentation microbiota in the early stage of production, whereas lactic acid and pH significantly correlated with the fermentation microbiota in the late stage of production (**Figure 2E**). In a previous study, to illustrate the variety of structures and functions within the fermentation microbiota, the fermentation process was separated into several stages (Tao et al., 2014). According to this report, liquor production could be separated into two distinct stages. Stage I was a period of high-level alcohol production in the samples from days 0 to 15. During this stage, the ethanol content increased quickly from 18.87 ± 14.98 g/kg to 37.03 ± 16.36 g/kg fermented grain. The species richness and diversity within the bacteria microbiota decreased, whereas diversity within the fungal microbiota increased (Supplementary Tables S1, S2). Stage II was a period of high-level acid production in the 30.59 ± 6.37 g/kg to 36.20 ± 6.21 g/kg fermented grain. The species richness and diversity of the bacterial microbiota decreased, whereas that of the fungal microbiota remained unchanged during the second stage (Supplementary Tables S1, S2).

As a result of the complex endogenous factors present in a closed system (i.e., the pit fermentation process), it is a challenge to link the core microbiota to major flavor metabolites (such as alcohols and acids) in liquor production (Supplementary Figure S1). Many studies had described the distribution of species in the microbiota and possible correlations between the microbiota and products in different environmental conditions, but these studies have not identified the critical metabolic pathways involved (Graham et al., 2014; Wang Z. et al., 2014). Therefore, in our study, amplicons and metatranscriptomic data were combined to investigate potential correlations between microorganisms, functions and products. In our study, the core bacteria (genera *Lactobacillus* and *Kroppenstedtia*) and core fungi (genera *Saccharomyces, Schizosaccharomyces, Zygosaccharomyces*, and *Saccharomycopsis*) were significantly correlated with major endogenous factors at different stages of liquor production (*P* < 0.05) (**Figure 2**). Moreover, metatranscriptomic analysis indicated the relative abundance of unigene expression related to glycolysis/gluconeogenesis, the TCA cycle and pyruvate metabolism increased from 6.79 to 8.24% in the microbiota (Supplementary Figure S5). In addition, KEGG data illustrated that the bacterial genus (*Lactobacillus*) and fungal genera (*Schizosaccharomyces, Pichia, Zygosaccharomyces*, and *Saccharomyces*) were the core functional microbiota in liquor production (Supplementary Figure S6). These results showed that yeasts (genera *Schizosaccharomyces, Pichia, Zygosaccharomyces*, and *Saccharomyces*) and LAB (genus *Lactobacillus*) were dominant functional contributors to major alcohol and acid production (ethanol, lactic acid, and acetic acid production).

Accordingly, pyruvate metabolism related to ethanol, lactic acid, and acetic acid production in yeasts (genera *Pichia, Saccharomyces, Schizosaccharomyces*, and *Zygosaccharomyces*) and LAB (genus *Lactobacillus*) was selected to illustrate the functional shift (functional conversion from ethanol to lactic acid production) in different stages of liquor production (**Figures 4, 5**). Yeasts (genera *Pichia, Saccharomyces, Schizosaccharomyces*, and *Zygosaccharomyces*) in Stage I expressed high levels of genes related to pyruvate metabolism. The genera *Pichia, Saccharomyces*, and *Zygosaccharomyces* were facultative aerobic microorganisms that were separated from food or wines (Pitt and Hocking, 2009). They were able to grow aerobically or anaerobically at low pH and had the ability to limit ethanol and acetic acid production (Kawahata et al., 2006; Yuangsaard et al., 2013; Lindahl et al., 2016). During this stage, the genera *Pichia, Saccharomyces*, and *Zygosaccharomyces* converted lactic acid to pyruvate. Moreover, they even converted pyruvate to acetyl-CoA, acetaldehyde in liquor production (**Figures 4, 5D**). Acetyl-CoA is an important intermediate metabolite from the conversion of pyruvate to acetic acid under aerobic or anaerobic conditions (Edirisinghe et al., 2016). The genes PDH E1, ACS, ALDH, and ADH encode a series of consecutively used enzymes that convert pyruvate to ethanol via acetyl-CoA, acetate, and acetaldehyde. With increasing levels of the genus *Schizosaccharomyces* as the main contributor under aerobic conditions and high moisture, ethanol production was the major microbial function in Stage I (**Figure 5C**). At this stage, the ACS, PYC, and ALDH genes were not expressed in the genus *Lactobacillus* (**Figure 4**). Although the genes related to pyruvate oxidase (POXL) might be expressed at high levels, but it had not activity at anaerobic conditions in liquor production. Generally, enzymes related to pyruvate metabolism were highly expressed in yeasts, but were expressed at low-level in the genus *Lactobacillus* (**Figure 5**). In addition, the genus *Lactobacillus* in Stage II expressed high levels of the genes related to pyruvate metabolism. The genus *Lactobacillus* comprises facultative anaerobic microorganisms identified from cheeses or wines (Lahtinen et al., 2012; Su et al., 2014). This genus was able to grow anaerobically at low pH and had the ability to adapt to a large temperature variation (10–45°C) (Fröhlich-Wyder et al., 2015; Johanningsmeier and McFeeters, 2015; Wang et al., 2015). During this stage, the genus *Lactobacillus* increased lactic acid productivity and maintained ethanol productivity simultaneously (**Figure 4**). LDH in the genus *Lactobacillus* converted pyruvate to lactic acid, while at the same time, XFP in the genus *Lactobacillus* converted glucose to acetyl phosphate directly in heterofermentative fermentation (**Figures 4, 5E,H**). With the genus *Lactobacillus* being the main contributor under low pH, lactic acid production was the major microbial function in Stage II (**Figure 5F**).

It was intriguing to understand why the genera *Schizosaccharomyces* and *Lactobacillus* were the major functional contributors among the core microbiota. With the initiation of ethanol production in Stage I, the resulting high ethanol content may inhibit some core bacteria (genera *Bacillus, Acetobacter*, and *Pediococcus*) in the microbiota (Araque et al., 2013; Kim et al., 2013; Sowards et al., 2014). Similarly, with the increase in lactic acid content in Stage II, the high content of lactic acid may inhibit some core yeasts (genera *Pichia, Schizosaccharomyces*, and *Zygosaccharomyces*) suppressing ethanol production by these genera in the microbiota (Crowley et al., 2013; Su et al., 2014). Our study found that the genes of four key enzymes in heterolactic fermentation and three key enzymes in homolactic fermentation were expressed (Supplementary Figure S7). Besides, the gene expressions of four key enzymes in heterolactic fermentation were much higher than three key enzymes in homolactic fermentation. This phenomenon showed that the most *Lactobacillus* species had ability to produce lactic acid, ethanol and acetic acid. Previous research showed that *L. acidophilus, L. delbrueckii, L*. *helveticus*, and *L. salivarius* were homofermentative lactobacilli. Except these, most *Lactobacillus* genera were facultative or obligatory heterofermentative lactobacilli (Lahtinen et al., 2012; Gänzle, 2015). In Supplementary Figures S8, S9, homofermentative and heterofermentative metabolism in the genus *Lactobacillus* showed that many lactobacilli played their significant function in two-type metabolism and most lactobacilli were facultative or obligatory heterofermentative lactobacilli in Chinese soy sauce aroma liquor production. Therefore, the genus *Lactobacillus* had developed several survival systems in various acidic environments to prevent cell damage resulting from acid stress by the acid product during the fermentation process (Liu et al., 2015). In addition, a variety of heterofermentative *Lactobacillus* genera had characteristics about rapid growth (Tamarit et al., 2015) and effective carbohydrate metabolism (Gänzle and Ripari, 2016). Moreover, some homofermentative *Lactobacillus* genera could produce antibacterial peptides and bacteriocins that enable these organisms to compete against other bacteria in the microbiota, which confers a competitive advantage over other microorganisms in many food industries by reducing the risks of food poisoning and protecting human health against food spoilage (Messaoudi et al., 2013; Garrote et al., 2015). Furthermore, the genus *Lactobacillus* exerts antifungal effects on food or feed-borne filamentous fungi and yeasts in traditional SSF, such as kimchi fermentation (Ryu et al., 2014). For the reasons stated above, the genus *Lactobacillus* became the predominant microorganism in liquor production and the major lactic acid and acetic acid producer under strict anaerobic conditions during liquor production. However, the acetic acid content decreased between Stages I and II (**Figure 5I**), for which there are several possible reasons. Firstly, low expression of the acetate kinase (ACK, K00925) and ACS gene might indicate that it encodes the key regulatory enzyme in acetic acid production. Secondly, ethyl acetate was the most abundant ester among the flavor components, the synthesis of which required large amounts of ethanol and acetic acid, thus decreasing the acetic acid content (Lin et al., 2012). Therefore, acetic acid was still responsible for acid production in Stage II and the genus *Lactobacillus* was still the microbial indicator of the major functional shift from Stage I to Stage II in liquor production. As suggested above, a variety of core functional microbiota can be attributed to the different stages of liquor production. Although 11 bacterial and 10 fungal genera were recognized as the core microbiota, only the genera *Schizosaccharomyces* and *Lactobacillus* possessed the metabolic capacity to form ethanol, lactic acid and acetic acid in the flavor metabolites.

In our study, we unveiled the structure and function of the core microbiota in Chinese soy sauce aroma type liquor production by high-throughput sequencing technologies and identified the type and quantity of flavor components by UPLC and HS-SPME-GC-MS. Amplicon analysis revealed fundamental information about microbial succession and the positive correlations between the core microbiota structure and major endogenous factors. Furthermore, metatranscriptomic database analysis demonstrated a large amount of information regarding the variety of core functional contributors in the fermentation microbiota, thus making it possible to correlate the core functional microbiota with important flavor metabolites. Finally, we identified many microorganisms as members of the core structural microbiota that had the metabolic capacity to perform specific functional roles. Only a few microorganisms, constituting the core functional microbiota, played a critical role in driving the variety of microbiota. According to the best of our knowledge, this is the first report to analyze the core microbiota in Chinese soy sauce aroma type liquor production systemically and to define the core functional microbiota in different fermentation processes. These findings enrich our knowledge of the core functional microbiota in the fermentation process under artificial conditions or natural environments.

## Author Contributions

ZS, HD, YZ, and YX designed this research. ZS, HD, YZ, and YX executed the experiments and analyzed the data. ZS wrote the paper.

## Conflict of Interest Statement

The authors declare that the research was conducted in the absence of any commercial or financial relationships that could be construed as a potential conflict of interest.

## References

[B1] Aldrete-Tapia A. Escobar-Ramírez M. C. Tamplin M. L. Hernández-Iturriaga M. (2014). High-throughput sequencing of microbial communities in Poro cheese, an artisanal Mexican cheese. *Food Microbiol.* 44 136–141. 10.1016/j.fm.2014.05.022 25084655

[B2] Angly F. E. Heath C. Morgan T. C. Tonin H. Rich V. Schaffelke B. (2016). Marine microbial communities of the Great Barrier Reef lagoon are influenced by riverine floodwaters and seasonal weather events. *Peer J.* 4:e1511 10.7717/peerj.1511 PMC4734448 26839738

[B3] Araque I. Bordons A. Reguant C. (2013). Effect of ethanol and low pH on citrulline and ornithine excretion and arc gene expression by strains of *Lactobacillus brevis* and *Pediococcus pentosaceus*. *Food Microbiol.* 33 107–113. 10.1016/j.fm.2012.09.005 23122508

[B4] Astudillo-Garcia C. Bell J. J. Webster N. S. Glasl B. Jompa J. Montoya J. M. (2017). Evaluating the core microbiota in complex communities: a systematic investigation. *Environ. Microbiol.* 19 1450–1462. 10.1111/1462-2920.13647 28078754

[B5] Azwar M. Y. Hussain M. A. Abdul-Wahab A. K. (2014). Development of biohydrogen production by photobiological, fermentation and electrochemical processes: a review. *Renew. Sustain. Energy Rev.* 31 158–173. 10.1016/j.rser.2013.11.022

[B6] Bokulich N. A. Mills D. A. (2012). Next-generation approaches to the microbial ecology of food fermentations. *BMB Rep.* 45 377–389. 10.5483/BMBRep.2012.45.7.148 22831972

[B7] Bolger A. M. Lohse M. Usadel B. (2014). Trimmomatic: a flexible trimmer for Illumina sequence data. *Bioinformatics* 30 2114–2120. 10.1093/bioinformatics/btu170 24695404 PMC4103590

[B8] Borkowf C. B. (2002). Computing the nonnull asymptotic variance and the asymptotic relative efficiency of Spearman’s rank correlation. *Comput. Stat. Data Anal.* 39 271–286. 10.1016/S0167-9473(01)00081-0

[B9] Buchfink B. Xie C. Huson D. H. (2015). Fast and sensitive protein alignment using DIAMOND. *Nat. Methods* 12 59–60. 10.1038/nmeth.3176 25402007

[B10] Caporaso J. G. Kuczynski J. Stombaugh J. Bittinger K. Bushman F. D. Costello E. K. (2010). QIIME allows analysis of high-throughput microbiota sequencing data. *Nat. Med.* 7 335–336. 10.1038/nmeth.f.303 PMC3156573 20383131

[B11] Chen B. Wu Q. Xu Y. (2014). Filamentous fungal diversity and community structure associated with the solid state fermentation of Chinese Maotai-flavor liquor. *Int. J. Food Microbiol.* 179 80–84. 10.1016/j.ijfoodmicro.2014.03.011 24742997

[B12] Chen Y. Wen Y. Tang Z. Huang J. Zhou Q. Vymazal J. (2015). Effects of plant biomass on bacterial microbiota structure in constructed wetlands used for tertiary wastewater treatment. *Ecol. Eng.* 84 38–45. 10.1016/j.ecoleng.2015.07.013

[B13] Cocolin L. Alessandria V. Dolci P. Gorra R. Rantsiou K. (2013). Culture independent methods to assess the diversity and dynamics of microbiota during food fermentation. *Int. J. Food Microbiol.* 167 29–43. 10.1016/j.ijfoodmicro.2013.05.008 23791362

[B14] Cordovez V. Carrion V. J. Etalo D. W. Mumm R. Zhu H. Van Wezel G. P. (2015). Diversity and functions of volatile organic compounds produced by Streptomyces from a disease-suppressive soil. *Front. Microbiol.* 6:1081 10.3389/fmicb.2015.01081 PMC4598592 26500626

[B15] Crowley S. Mahony J. van Sinderen D. (2013). Current perspectives on antifungal lactic acid bacteria as natural bio-preservatives. *Trends Food Sci. Technol.* 33 93–109. 10.1016/j.tifs.2013.07.004

[B16] De Filippis F. Genovese A. Ferranti P. Gilbert J. A. Ercolini D. (2016). Metatranscriptomics reveals temperature-driven functional changes in microbiome impacting cheese maturation rate. *Sci. Rep.* 6:21871 10.1038/srep21871 PMC4766472 26911915

[B17] Deng W. Wang Y. Liu Z. Cheng H. Xue Y. (2014). HemI: a toolkit for illustrating heatmaps. *PLoS ONE* 9:e111988 10.1371/journal.pone.0111988 PMC4221433 25372567

[B18] Di Cagno R. Pontonio E. Buchin S. De Angelis M. Lattanzi A. Valerio F. (2014). Diversity of the lactic acid bacteria and yeast microbiota switching from firm to liquid sourdough fermentation. *Appl. Environ. Microbiol.* 80 3161–3172. 10.1128/AEM.00309-14 24632249 PMC4018931

[B19] Edirisinghe J. N. Weisenhorn P. Conrad N. Xia F. Overbeek R. Stevens R. L. (2016). Modeling central metabolism and energy biosynthesis across microbial life. *BMC Genomics* 17:568 10.1186/s12864-016-2887-8 PMC4977884 27502787

[B20] Ercolini D. (2013). High-throughput sequencing and metagenomics: moving forward in the culture-independent analysis of food microbial ecology. *Appl. Environ. Microbiol.* 79 3148–3155. 10.1128/AEM.00928-13 23475615 PMC3685257

[B21] Fierer N. Ladau J. Clemente J. C. Leff J. W. Owens S. M. Pollard K. S. (2013). Reconstructing the microbial diversity and function of pre-agricultural tallgrass prairie soils in the United States. *Science* 342 621–624. 10.1126/science.1243768 24179225

[B22] Fröhlich-Wyder M. T. Bisig W. Guggisberg D. Irmler S. Jakob E. Wechsler D. (2015). Influence of low pH on the metabolic activity of *Lactobacillus buchneri* and *Lactobacillus parabuchneri* strains in Tilsit-type model cheese. *Dairy Sci. Technol.* 95 569–585. 10.1007/s13594-015-0238-1

[B23] Gänzle M. Ripari V. (2016). Composition and function of sourdough microbiota: from ecological theory to bread quality. *Int. J. Food Microbiol.* 239 19–25. 10.1016/j.ijfoodmicro.2016.05.004 27240932

[B24] Gänzle M. G. (2015). Lactic metabolism revisited: metabolism of lactic acid bacteria in food fermentations and food spoilage. *Curr. Opin. Food Sci.* 2 106–117. 10.1016/j.cofs.2015.03.001

[B25] Garrote G. L. Abraham A. G. Rumbo M. (2015). Is lactate an undervalued functional component of fermented food products? *Front. Microbiol.* 6:629 10.3389/fmicb.2015.00629 PMC4473639 26150815

[B26] Giraffa G. (2004). Studying the dynamics of microbial populations during food fermentation. *FEMS Microbiol. Rev.* 28 251–260. 10.1016/j.femsre.2003.10.005 15109787

[B27] Glass J. B. Kretz C. B. Ganesh S. Ranjan P. Seston S. L. Buck K. N. (2015). Meta-omic signatures of microbial metal and nitrogen cycling in marine oxygen minimum zones. *Front. Microbiol.* 6:998 10.3389/fmicb.2015.00998 PMC4585252 26441925

[B28] Graham E. B. Wieder W. R. Leff J. W. Weintraub S. R. Townsend A. R. Cleveland C. C. (2014). Do we need to understand microbial communities to predict ecosystem function? A comparison of statistical models of nitrogen cycling processes. *Soil Biol. Biochem.* 68 279–282. 10.1016/j.soilbio.2013.08.023

[B29] Gulati A. Swarnkar M. K. Vyas P. Rahi P. Thakur R. Thakur N. (2015). Complete genome sequence of the rhizobacterium *Pseudomonas trivialis* strain IHBB745 with multiple plant growth-promoting activities and tolerance to desiccation and alkalinity. *Genome Announc.* 3:e00943-15 10.1128/genomeA.00943-15 PMC4559727 26337878

[B30] Guo Y. Li C. I. Ye F. Shyr Y. (2013). Evaluation of read count based RNAseq analysis methods. *BMC Genomics* 14:S2 10.1186/1471-2164-14-S8-S2 PMC4092879 24564449

[B31] Haas B. J. Papanicolaou A. Yassour M. Grabherr M. Blood P. D. Bowden J. (2013). De novo transcript sequence reconstruction from RNA-seq using the Trinity platform for reference generation and analysis. *Nat. Protoc.* 8 1494–1512. 10.1038/nprot.2013.084 23845962 PMC3875132

[B32] Hallin P. F. Ussery D. W. (2004). CBS Genome Atlas Database: a dynamic storage for bioinformatic results and sequence data. *Bioinformatics* 20 3682–3686. 10.1093/bioinformatics/bth423 15256401

[B33] Huson D. H. Auch A. F. Qi J. Schuster S. C. (2007). MEGAN analysis of metagenomic data. *Genome Res.* 17 377–386. 10.1101/gr.5969107 17255551 PMC1800929

[B34] Jacomy M. Venturini T. Heymann S. Bastian M. (2014). ForceAtlas2, a continuous graph layout algorithm for handy network visualization designed for the Gephi software. *PLoS ONE* 9:e98679 10.1371/journal.pone.0098679 PMC4051631 24914678

[B35] Johanningsmeier S. D. McFeeters R. F. (2015). Metabolic footprinting of *Lactobacillus buchneri* strain LA1147 during anaerobic spoilage of fermented cucumbers. *Int. J. Food Microbiol.* 215 40–48. 10.1016/j.ijfoodmicro.2015.08.004 26325599

[B36] Jung J. Y. Lee S. H. Jin H. M. Hahn Y. Madsen E. L. Jeon C. O. (2013). Metatranscriptomic analysis of lactic acid bacterial gene expression during kimchi fermentation. *Int. J. Food Microbiol.* 163 171–179. 10.1016/j.ijfoodmicro.2013.02.022 23558201

[B37] Kable M. E. Srisengfa Y. Laird M. Zaragoza J. McLeod J. Heidenreich J. (2016). The core and seasonal microbiota of raw bovine milk in tanker trucks and the impact of transfer to a milk processing facility. *MBio* 7:e00836-16 10.1128/mBio.00836-16 PMC4999540 27555305

[B38] Kanehisa M. Goto S. Kawashima S. Okuno Y. Hattori M. (2004). The KEGG resource for deciphering the genome. *Nucleic Acids Res.* 32 D277–D280. 10.1093/nar/gkh063 14681412 PMC308797

[B39] Kawahata M. Masaki K. Fujii T. Iefuji H. (2006). Yeast genes involved in response to lactic acid and acetic acid: acidic conditions caused by the organic acids in *Saccharomyces cerevisiae* cultures induce expression of intracellular metal metabolism genes regulated by Aft1p. *FEMS Yeast Res.* 6 924–936. 10.1111/j.1567-1364.2006.00089.x 16911514

[B40] Kim S. A. Lee M. K. Park T. H. Rhee M. S. (2013). A combined intervention using fermented ethanol and supercritical carbon dioxide to control *Bacillus cereus* and *Bacillus subtilis* in rice. *Food Control* 32 93–98. 10.1016/j.foodcont.2012.11.016

[B41] Kõljalg U. Nilsson R. H. Abarenkov K. Tedersoo L. Taylor A. F. Bahram M. (2013). Towards a unified paradigm for sequence-based identification of fungi. *Mol. Ecol.* 22 5271–5277. 10.1111/mec.12481 24112409

[B42] Kong Y. Wu Q. Zhang Y. Xu Y. (2014). In situ analysis of metabolic characteristics reveals the key yeast in the spontaneous and solid-state fermentation process of Chinese light-style liquor. *Appl. Environ. Microbiol.* 80 3667–3676. 10.1128/AEM.04219-13 24727269 PMC4054142

[B43] Lahtinen S. Ouwehand A. C. Salminen S. von Wright A. (eds) (2012). *Lactic Acid Bacteria: Microbiological and Functional Aspects* 4th Edn. New York, NY: CRC Press.

[B44] Lin Z. R. Zeng X. A. Yu S. J. Sun D. W. (2012). Enhancement of ethanol–acetic acid esterification under room temperature and non-catalytic condition via pulsed electric field application. *Food Bioprocess Technol.* 5 2637–2645. 10.1007/s11947-011-0678-4

[B45] Lindahl L. Genheden S. Eriksson L. A. Olsson L. Bettiga M. (2016). Sphingolipids contribute to acetic acid resistance in *Zygosaccharomyces bailii*. *Biotechnol. Bioeng.* 113 744–753. 10.1002/bit.25845 26416641 PMC5064642

[B46] Liu T. Zhu S. Tang Q. Chen P. Yu Y. Tang S. (2013). De novo assembly and characterization of transcriptome using Illumina paired-end sequencing and identification of CesA gene in ramie (*Boehmeria nivea* L. Gaud). *BMC Genomics* 14:125 10.1186/1471-2164-14-125 PMC3610122 23442184

[B47] Liu Y. Tang H. Lin Z. Xu P. (2015). Mechanisms of acid tolerance in bacteria and prospects in biotechnology and bioremediation. *Biotechnol. Adv.* 33 1484–1492. 10.1016/j.biotechadv.2015.06.001 26057689

[B48] Long Y. Yi H. Chen S. Zhang Z. Cui K. Bing Y. (2016a). Influences of plant type on bacterial and archaeal communities in constructed wetland treating polluted river water. *Environ. Sci. Pollut. Res. Int.* 23 19570–19579. 10.1007/s11356-016-7166-3 27392623

[B49] Long Y. Zhang Z. Pan X. Li B. Xie S. Guo Q. (2016b). Substrate influences on archaeal and bacterial assemblages in constructed wetland microcosms. *Ecol. Eng.* 94 437–442. 10.1016/j.ecoleng.2016.06.015

[B50] Lu Z. M. Liu N. Wang L. J. Wu L. H. Gong J. S. Yu Y. J. (2016). Elucidating and regulating the acetoin production role of microbial functional groups in multispecies acetic acid fermentation. *Appl. Environ. Microbiol.* 82 5860–5868. 10.1128/AEM.01331-16 27451452 PMC5038022

[B51] McGovern P. E. Zhang J. Tang J. Zhang Z. Hall G. R. Moreau R. A. (2004). Fermented beverages of pre-and proto-historic China. *Proc. Natl. Acad. Sci. U.S.A.* 101 17593–17598. 10.1073/pnas.0407921102 15590771 PMC539767

[B52] Messaoudi S. Manai M. Kergourlay G. Prévost H. Connil N. Chobert J. M. (2013). *Lactobacillus salivarius*: bacteriocin and probiotic activity. *Food Microbiol.* 36 296–304. 10.1016/j.fm.2013.05.010 24010610

[B53] Papagianni M. (2014). Recent advances in solid-state fermentation applications for the food industry. *Curr. Biochem. Eng.* 1 2–8.

[B54] Papalexandratou Z. Lefeber T. Bahrim B. Lee O. S. Daniel H. M. De Vuyst L. (2013). *Hanseniaspora opuntiae, Saccharomyces cerevisiae, Lactobacillus fermentum*, and *Acetobacter pasteurianus* predominate during well-performed Malaysian cocoa bean box fermentations, underlining the importance of these microbial species for a successful cocoa bean fermentation process. *Food Microbiol.* 35 73–85. 10.1016/j.fm.2013.02.015 23664257

[B55] Pitt J. I. Hocking A. D. (2009). *Fungi and Food Spoilage.* London: Springer.

[B56] Qi Z. Yang H. Xia X. Wang W. Yu X. (2014). High strength vinegar fermentation by *Acetobacter pasteurianus* via enhancing alcohol respiratory chain. *Biotechnol. Bioprocess Eng.* 19 289–297. 10.1007/s12257-013-0727-0

[B57] Rantsiou K. Cocolin L. (2006). New developments in the study of the microbiota of naturally fermented sausages as determined by molecular methods: a review. *Int. J. Food Microbiol.* 108 255–267. 10.1016/j.ijfoodmicro.2005.11.013 16481061

[B58] Ricciardi A. Guidone A. Ianniello R. G. Cioffi S. Aponte M. Pavlidis D. (2015). A survey of non-starter lactic acid bacteria in traditional cheeses: culture dependent identification and survival to simulated gastrointestinal transit. *Int. Dairy J.* 43 42–50. 10.1016/j.idairyj.2014.11.006

[B59] Ryu E. H. Yang E. J. Woo E. R. Chang H. C. (2014). Purification and characterization of antifungal compounds from *Lactobacillus plantarum* HD1 isolated from kimchi. *Food Microbiol.* 41 19–26. 10.1016/j.fm.2014.01.011 24750809

[B60] Schmitt S. Tsai P. Bell J. Fromont J. Ilan M. Lindquist N. (2012). Assessing the complex sponge microbiota: core, variable and species-specific bacterial communities in marine sponges. *ISME J.* 6 564–576. 10.1038/ismej.2011.116 21993395 PMC3280146

[B61] Schoch C. L. Seifert K. A. Huhndorf S. Robert V. Spouge J. L. Levesque C. A. (2012). Nuclear ribosomal internal transcribed spacer (ITS) region as a universal DNA barcode marker for Fungi. *Proc. Natl. Acad. Sci. U.S.A* 109 6241–6246. 10.1073/pnas.1117018109 22454494 PMC3341068

[B62] Sekwati-Monang B. Valcheva R. Gänzle M. G. (2012). Microbial ecology of sorghum sourdoughs: effect of substrate supply and phenolic compounds on composition of fermentation microbiota. *Int. J. Food Microbiol.* 159 240–246. 10.1016/j.ijfoodmicro.2012.09.013 23107503

[B63] Solieri L. Dakal T. C. Giudici P. (2013). Next-generation sequencing and its potential impact on food microbial genomics. *Ann. Microbiol.* 63 21–37. 10.1007/s13213-012-0478-8

[B64] Sowards J. W. Williamson C. H. D. Weeks T. S. McColskey J. D. Spear J. R. (2014). The effect of *Acetobacter* sp. and a sulfate-reducing bacterial consortium from ethanol fuel environments on fatigue crack propagation in pipeline and storage tank steels. *Corros. Sci.* 79 128–138. 10.1016/j.corsci.2013.10.036

[B65] Stegen J. C. Hurlbert A. H. Bond-Lamberty B. Chen X. Anderson C. G. Chu R. K. (2016). Aligning the measurement of microbial diversity with macroecological theory. *Front. Microbiol.* 7:1487 10.3389/fmicb.2016.01487 PMC5033968 27721808

[B66] Su J. Wang T. Wang Y. Li Y. Y. Li H. (2014). The use of lactic acid-producing, malic acid-producing, or malic acid-degrading yeast strains for acidity adjustment in the wine industry. *Appl. Microbiol. Biotechnol.* 98 2395–2413. 10.1007/s00253-014-5508-y 24430209

[B67] Sulaiman J. Gan H. M. Yin W. F. Chan K. G. (2014). Microbial succession and the functional potential during the fermentation of Chinese soy sauce brine. *Front. Microbiol.* 5:556 10.3389/fmicb.2014.00556 PMC4215829 25400624

[B68] Tamang J. P. Shin D. H. Jung S. J. Chae S. W. (2016). Functional properties of microorganisms in fermented foods. *Front. Microbiol.* 7:578 10.3389/fmicb.2016.00578 PMC4844621 27199913

[B69] Tamarit D. Ellegaard K. M. Wikander J. Olofsson T. Vásquez A. Andersson S. G. (2015). Functionally structured genomes in *Lactobacillus kunkeei* colonizing the honey crop and food products of honeybees and stingless bees. *Genome Biol. Evol.* 7 1455–1473. 10.1093/gbe/evv079 25953738 PMC4494060

[B70] Tao Y. Li J. Rui J. Xu Z. Zhou Y. Hu X. (2014). Prokaryotic communities in pit mud from different-aged cellars used for the production of Chinese strong-flavored liquor. *Appl. Environ. Microbiol.* 80 2254–2260. 10.1128/AEM.04070-13 24487528 PMC3993157

[B71] Thomas L. Larroche C. Pandey A. (2013). Current developments in solid-state fermentation. *Biochem. Eng. J.* 81 146–161. 10.1016/j.bej.2013.10.013

[B72] Wang S. Y. Ho Y. F. Chen Y. P. Chen M. J. (2015). Effects of a novel encapsulating technique on the temperature tolerance and anti-colitis activity of the probiotic bacterium *Lactobacillus kefiranofaciens* M1. *Food Microbiol.* 46 494–500. 10.1016/j.fm.2014.09.015 25475320

[B73] Wang X. Fan W. Xu Y. (2014). Comparison on aroma compounds in Chinese soy sauce and strong aroma type liquors by gas chromatography–olfactometry, chemical quantitative and odor activity values analysis. *Eur. Food Res. Technol.* 239 813–825. 10.1007/s00217-014-2275-z

[B74] Wang Z. Zhang X. X. Lu X. Liu B. Li Y. Long C. (2014). Abundance and diversity of bacterial nitrifiers and denitrifiers and their functional genes in tannery wastewater treatment plants revealed by high-throughput sequencing. *PLoS ONE* 9:e113603 10.1371/journal.pone.0113603 PMC4242629 25420093

[B75] Wang Z. M. Lu Z. M. Shi J. S. Xu Z. H. (2016). Exploring flavour-producing core microbiota in multispecies solid-state fermentation of traditional Chinese vinegar. *Sci. Rep.* 6:26818 10.1038/srep26818 PMC4886211 27241188

[B76] Whiteson K. L. Lazarevic V. Tangomo-Bento M. Girard M. Maughan H. Pittet D. (2014). Noma affected children from Niger have distinct oral microbial communities based on high-throughput sequencing of 16S rRNA gene fragments. *PLoS Negl. Trop. Dis.* 8:e3240 10.1371/journal.pntd.0003240 PMC4256271 25474262

[B77] Wolfe B. E. Button J. E. Santarelli M. Dutton R. J. (2014). Cheese rind communities provide tractable systems for in situ and in vitro studies of microbial diversity. *Cell* 158 422–433. 10.1016/j.cell.2014.05.041 25036636 PMC4222527

[B78] Wu Q. Chen L. Xu Y. (2013). Yeast community associated with the solid state fermentation of traditional Chinese Maotai-flavor liquor. *Int. J. Food Microbiol.* 166 323–330. 10.1016/j.ijfoodmicro.2013.07.003 23978339

[B79] Wu Q. Xu Y. Chen L. (2012). Diversity of yeast species during fermentative process contributing to Chinese Maotai-flavour liquor making. *Lett. Appl. Microbiol.* 55 301–307. 10.1111/j.1472-765X.2012.03294.x 22862564

[B80] Xu Y. Ji K. (2012). “Moutai (Maotai): production and sensory properties,” in *The Alcoholic Beverages: Sensory Evaluation and Consumer Research* ed. Piggott J. (Sawston: Woodhead Publishing) 315–330.

[B81] Xu Y. Wang D. Fan W. L. Mu X. Q. Chen J. (2010). Traditional Chinese biotechnology. *Adv. Biochem. Eng. Biotechnol.* 122 189–233. 10.1007/10_2008_36 19888561

[B82] Yuangsaard N. Yongmanitchai W. Yamada M. Limtong S. (2013). Selection and characterization of a newly isolated thermotolerant *Pichia kudriavzevii* strain for ethanol production at high temperature from cassava starch hydrolysate. *Antonie Van Leeuwenhoek* 103 577–588. 10.1007/s10482-012-9842-8 23132277

[B83] Zhang J. Guo Z. Xue Z. Sun Z. Zhang M. Wang L. (2015). A phylo-functional core of gut microbiota in healthy young Chinese cohorts across lifestyles, geography and ethnicities. *ISME J.* 9 1979–1990. 10.1038/ismej.2015.11 25647347 PMC4542028

[B84] Zhu S. Lu X. Ji K. Guo K. Li Y. Wu C. (2007). Characterization of flavor compounds in Chinese liquor Moutai by comprehensive two-dimensional gas chromatography/time-of-flight mass spectrometry. *Anal. Chim. Acta* 597 340–348. 10.1128/AEM.00594-15 17683748

[B85] Zhu X. Siegert M. Yates M. D. Logan B. E. (2015). Alamethicin suppresses methanogenesis and promotes acetogenesis in bioelectrochemical systems. *Appl. Environ. Microbiol.* 81 3863–3868. 10.1016/j.aca.2007.07.007 25819972 PMC4421063

[B86] Ziesemer K. A. Mann A. E. Sankaranarayanan K. Schroeder H. Ozga A. T. Brandt B. W. (2015). Intrinsic challenges in ancient microbiome reconstruction using 16S rRNA gene amplification. *Sci. Rep.* 5:16498 10.1038/srep16498 PMC4643231 26563586

[B87] Zothanpuia A. K. P. Chandra P. Leo V. V. Mishra V. K. Kumar B. Singh B. P. (2017). Production of potent antimicrobial compounds from *Streptomyces cyaneofuscatus* associated with fresh water sediment. *Front. Microbiol.* 8:68 10.3389/fmicb.2017.00068 PMC5263160 28179900

