# Supplementary material for: Unraveling Core Functional Microbiota in Traditional Solid-State Fermentation by High-Throughput Amplicons and Metatranscriptomics Sequencing
Source: Front Microbiol. 2017 Jul 14;8:1294. doi: 10.3389/fmicb.2017.01294 (PMC5509801; doi:10.3389/fmicb.2017.01294)
Supplement: Supplementary file 1 [file Data_Sheet_1.docx]

# *Supplementary Material*

**Unravelling core functional microbiota in traditional solid-state fermentation by high-throughput amplicons and metatranscriptomics sequencing**

**Zhewei Song^1^, Hai Du^1^, Yan Zhang^2^ and Yan Xu^1^***

^1^State Key Laboratory of Food Science and Technology, Key Laboratory of Industrial Biotechnology of Ministry of Education, Synergetic Innovation Center of Food Safety and Nutrition, School of Biotechnology, Jiangnan University, 1800 Lihu Avenue, Wuxi, Jiangsu 214122, China.

^2^State Key Laboratory of Microbial Metabolism, Joint International Research Laboratory of Metabolic and Developmental Sciences, School of Life Sciences and Biotechnology; Ministry of Education Key Laboratory of Systems Biomedicine, Shanghai Center for Systems Biomedicine (SCSB), Shanghai Jiao Tong University, Shanghai, 200240, China.

***Correspondence:** Yan Xu: yxu@jiangnan.edu.cn

**This file includes:**

Supplementary Figures (Figure S1-S9)

Supplementary Tables (Table S1-S2)

**Other supplementary information for this manuscript includes the following:**

Supplementary Table 3 as an Excel file: Supplementary_Table_S3.xls in data sheet 2

Supplementary Table 4 as an Excel file: Supplementary_Table_S4.xls in data sheet 2

1. **Supplementary Figures and Tables**
   1. **Supplementary Figures**

**
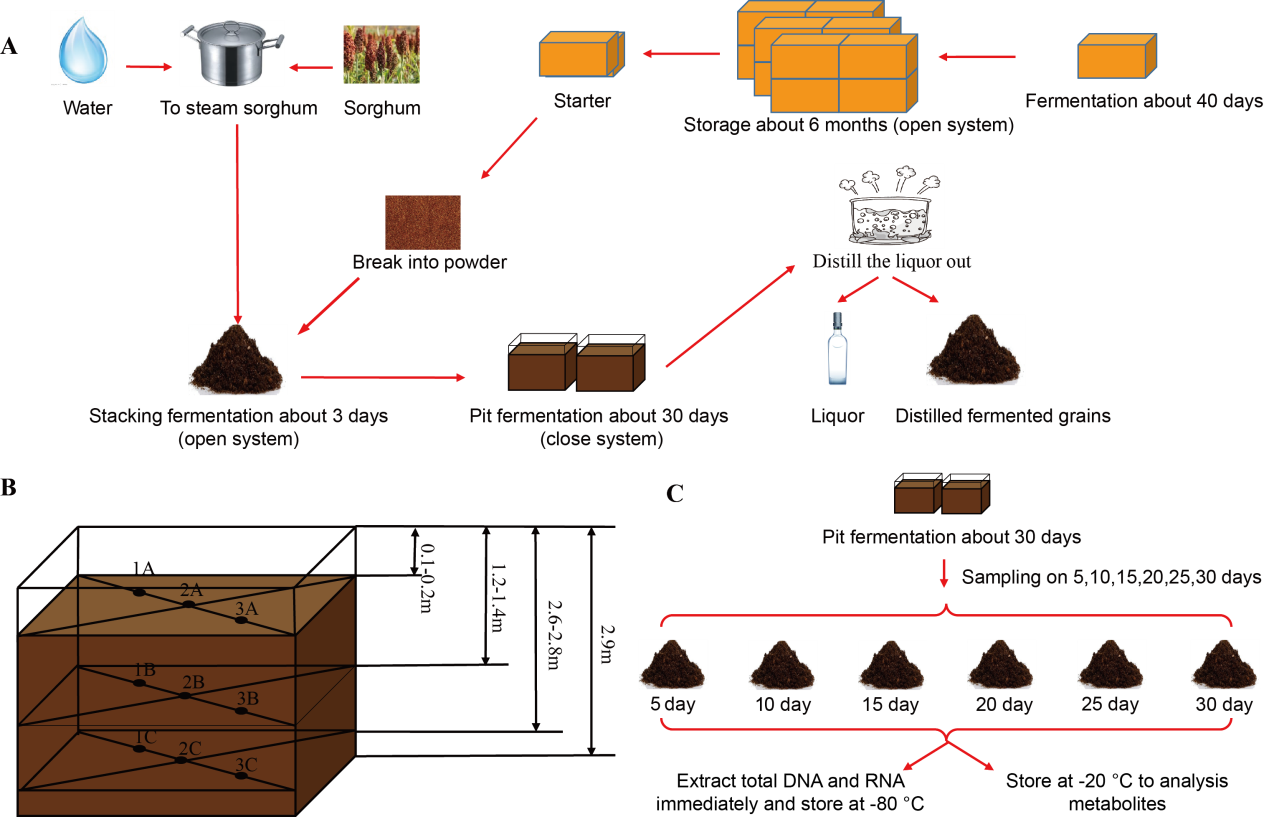
Figure S1.** Chinese Maotai-flavor liquor (CMFL) production. (A) A complete production involves different parts of the fermentation process. (B) The suffixes “A”, “B” and “C” indicated that samples were collected from the upper, middle and bottom layer in pit fermentation respectively. In addition, one final sample was made by mixing samples from three points (1, 2 and 3) in the same layer for reducing the volatility of samples. (C) Samples were collected at 5-day intervals in duplicate, one for genomic DNA extraction and total RNA extraction, the other for flavor component analysis.

**
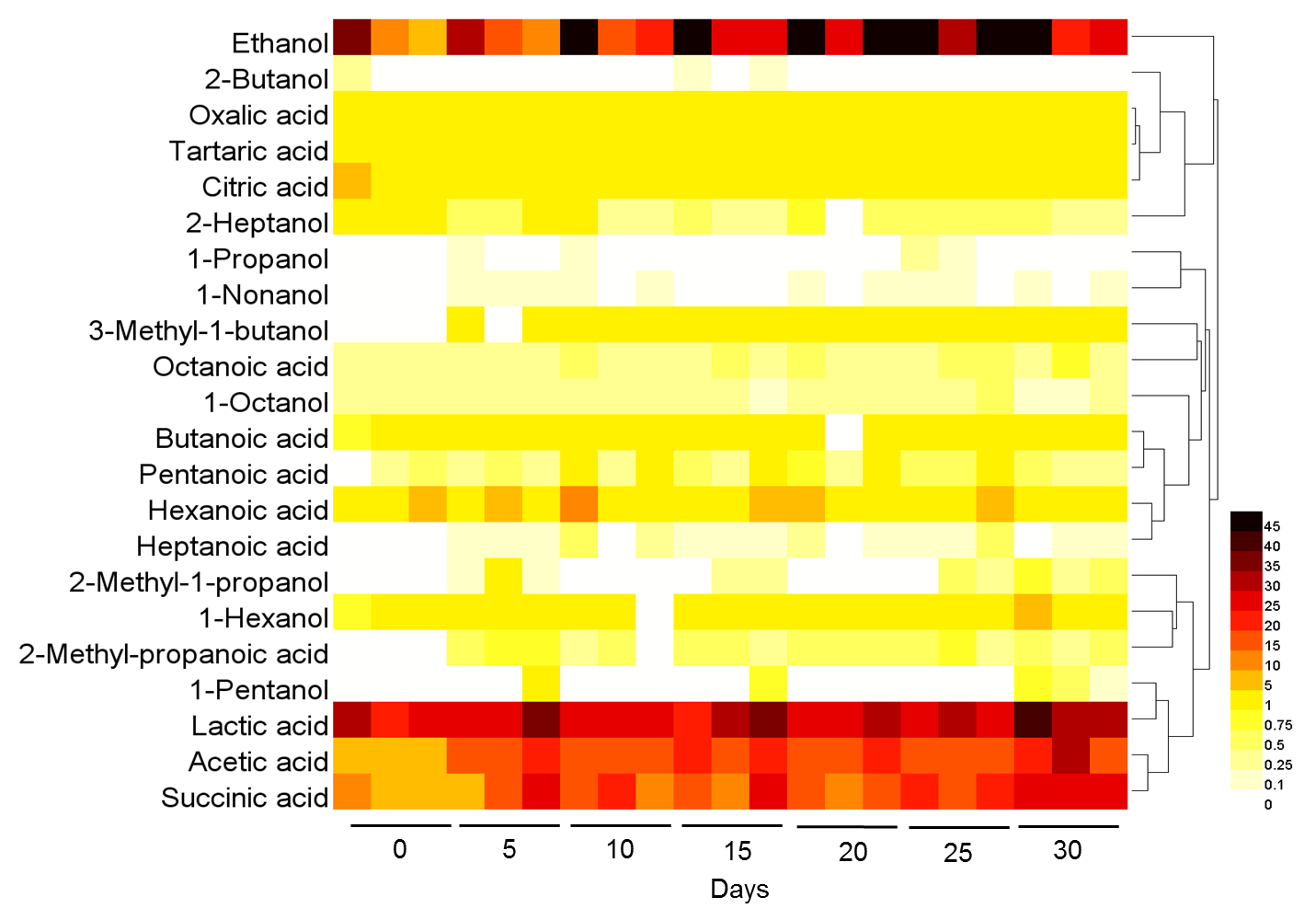
Figure S2.** The major flavor components (g/ kg fermented grain) in all samples (n = 21). Color depth represented the content of flavor components.


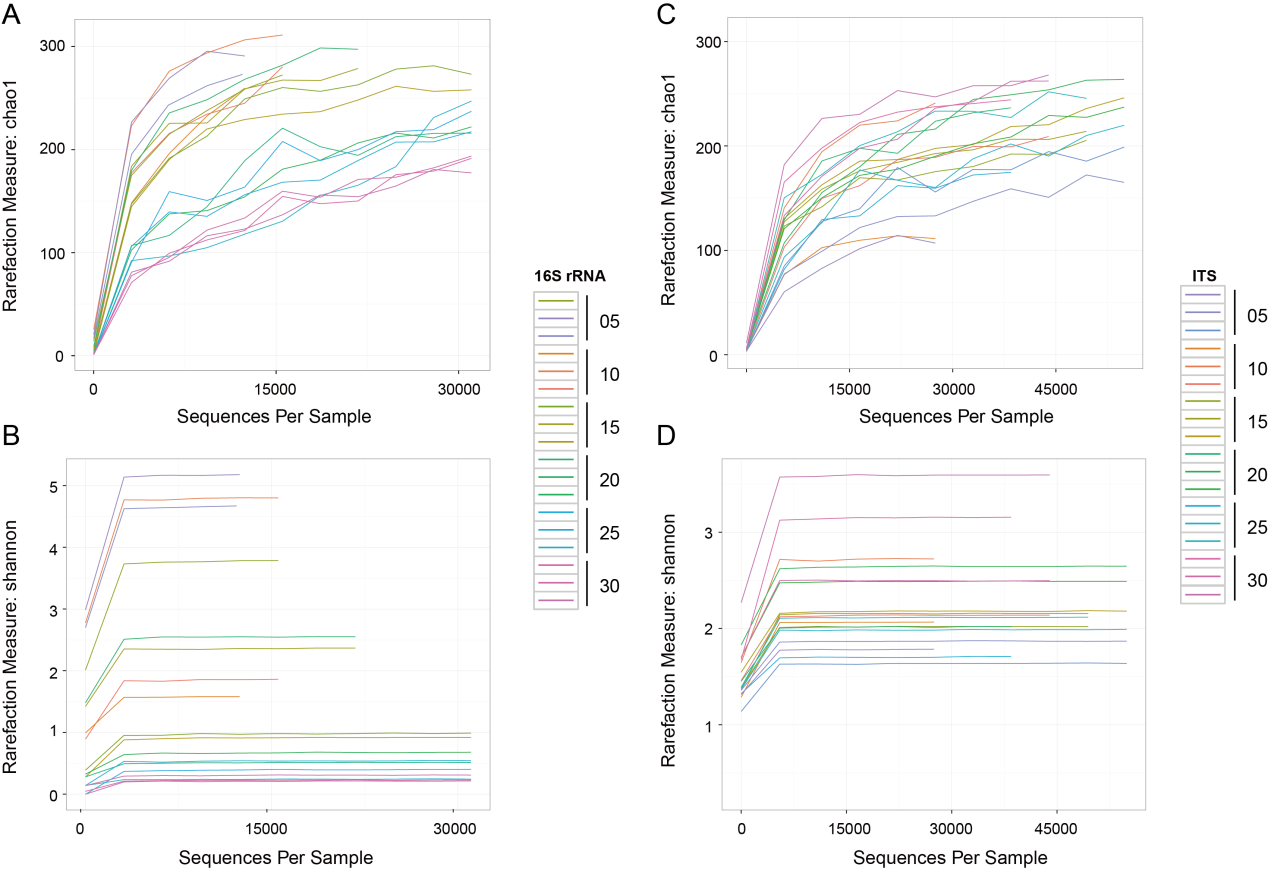


**Figure S3.** Comparison of the α-diversity indices for different methods. (A) Rarefaction curves of *Shannon* indices for 16S rRNA amplicons in all samples (n=18).(B) Rarefaction curves of *Chao1* indices for 16S rRNA amplicons in all samples (n=18). (C) Rarefaction curves of *Shannon* indices for ITS amplicons in all samples (n=18). (D) Rarefaction curves of *Chao1* indices for ITS amplicons in all samples (n=18).


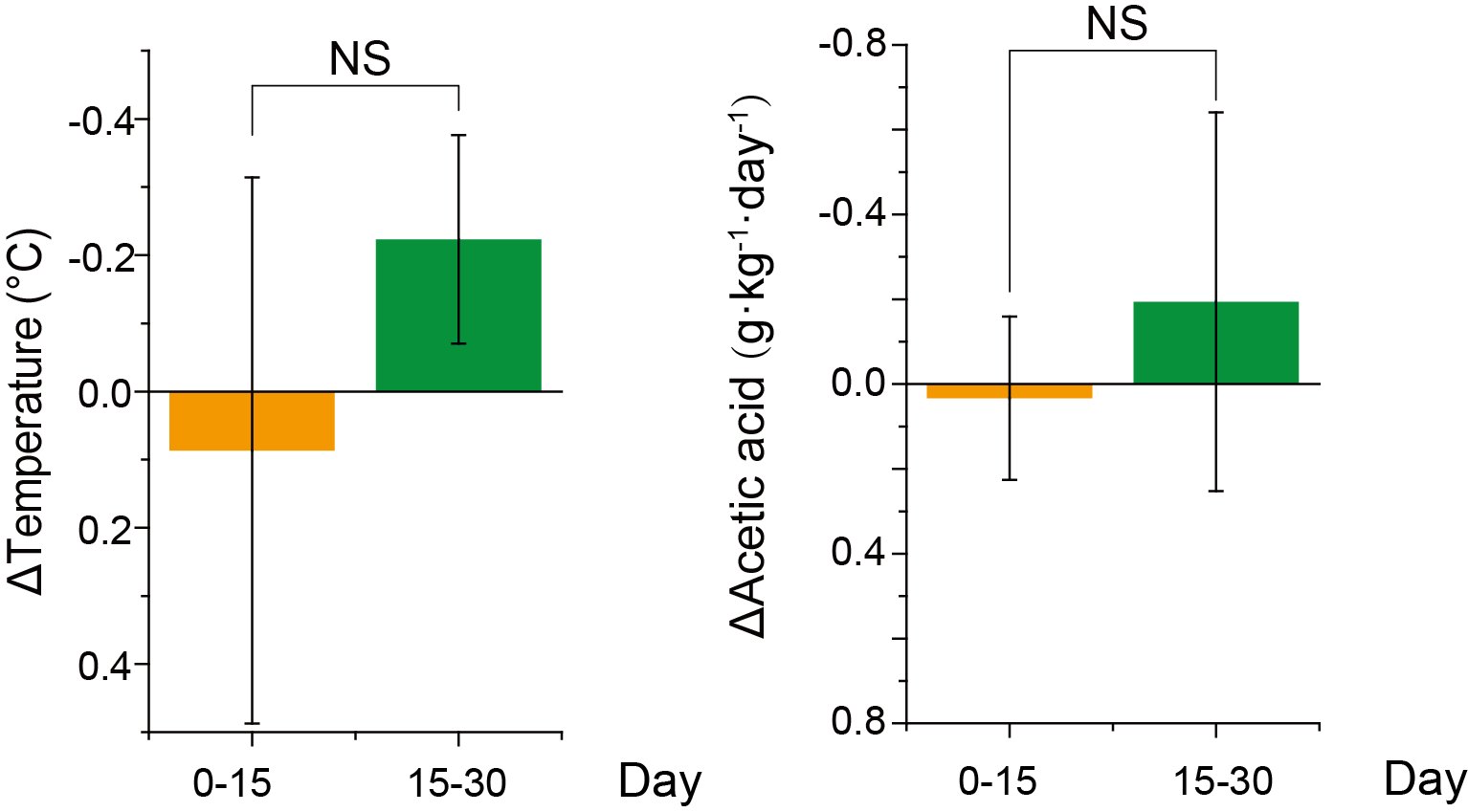


**Figure S4.** The relationship between environmental factors with microbita in CMFL production. The temperature and acetic acid were considered as the major environmental factors to effect the production of CMFL. Bars represent mean (± SE). Asterisk indicates significant differences (p < 0.01) in t-test. NS indicates not significant (p > 0.05).

**
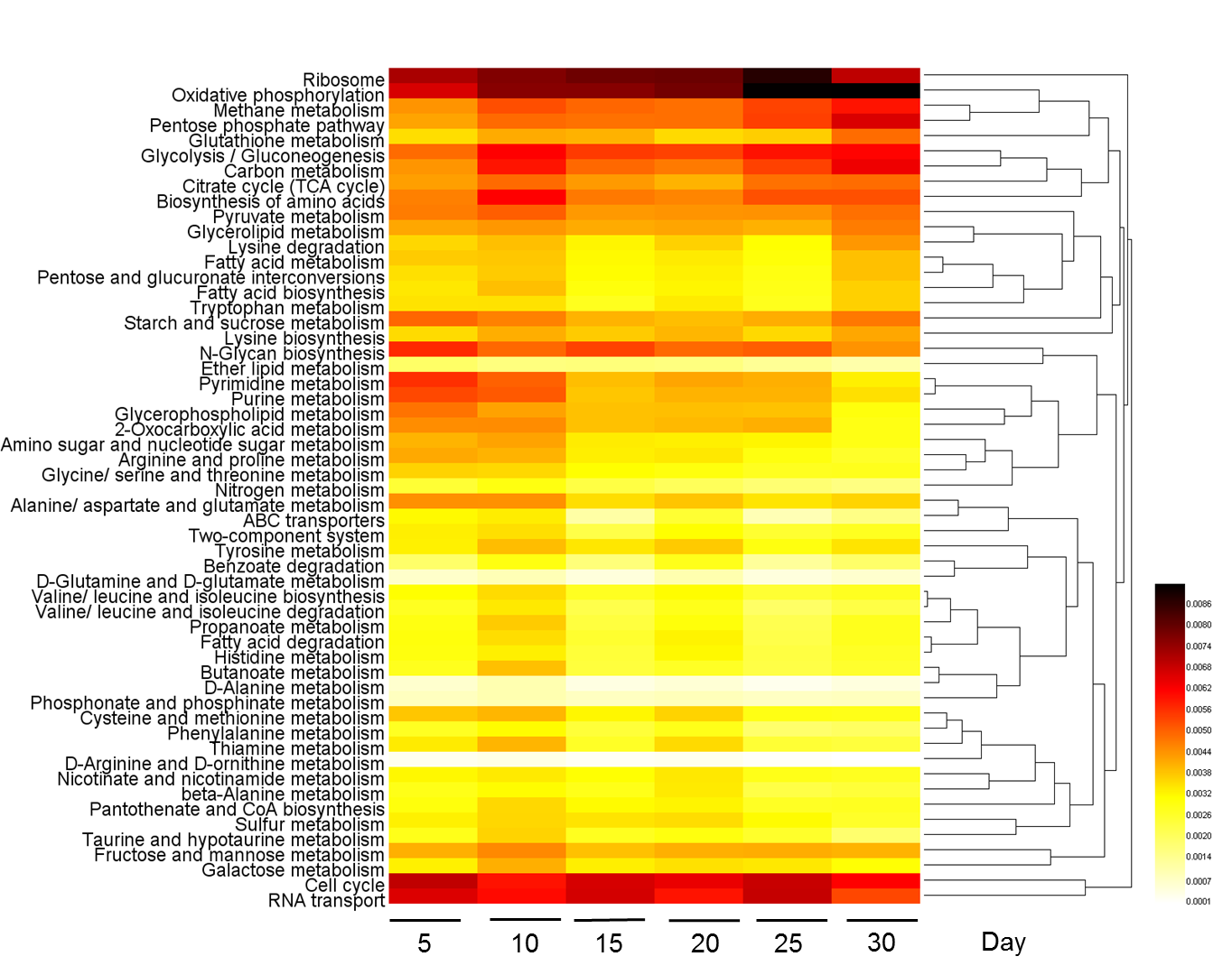
 Figure S5.** Relative abundance of partly expressed unigenes with KEGG metabolic pathways in samples (n = 6).

**
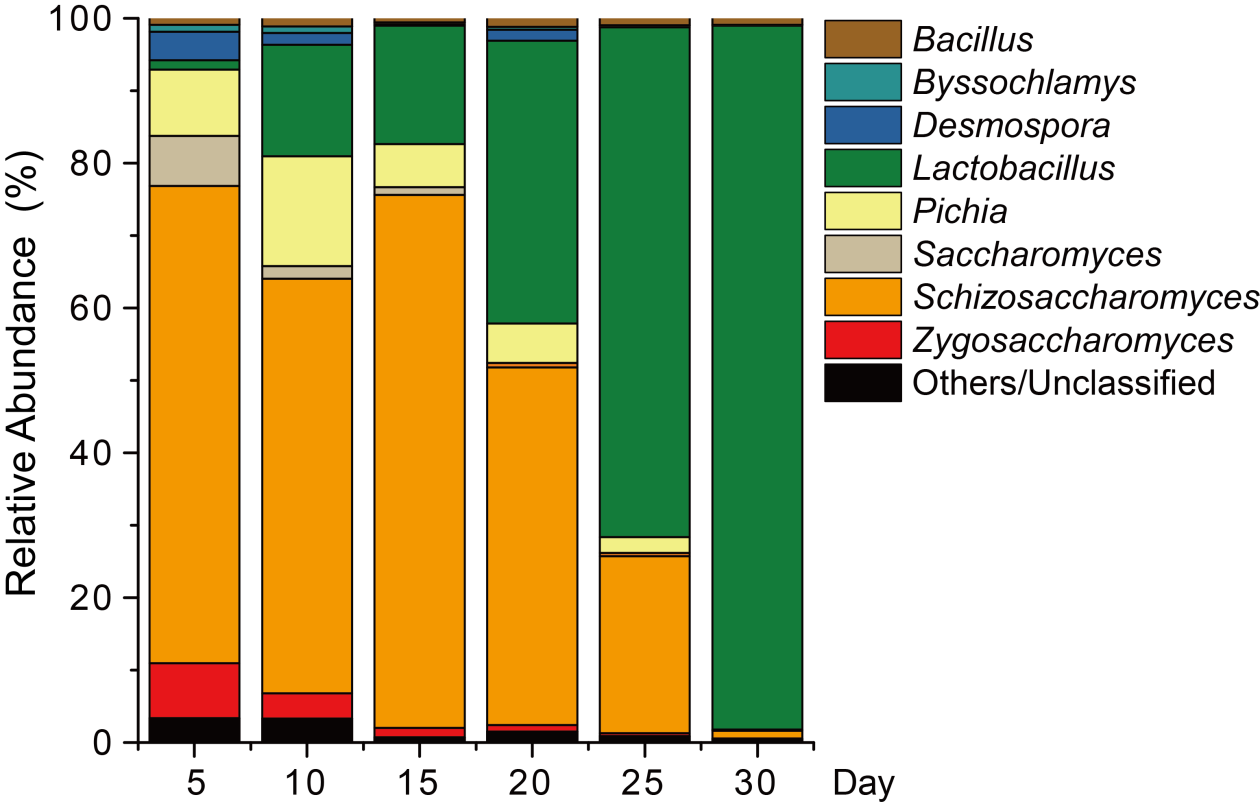
Figure S6.** Relative abundance of major functional microbiota in KEGG metabolic pathways in samples (n = 6).


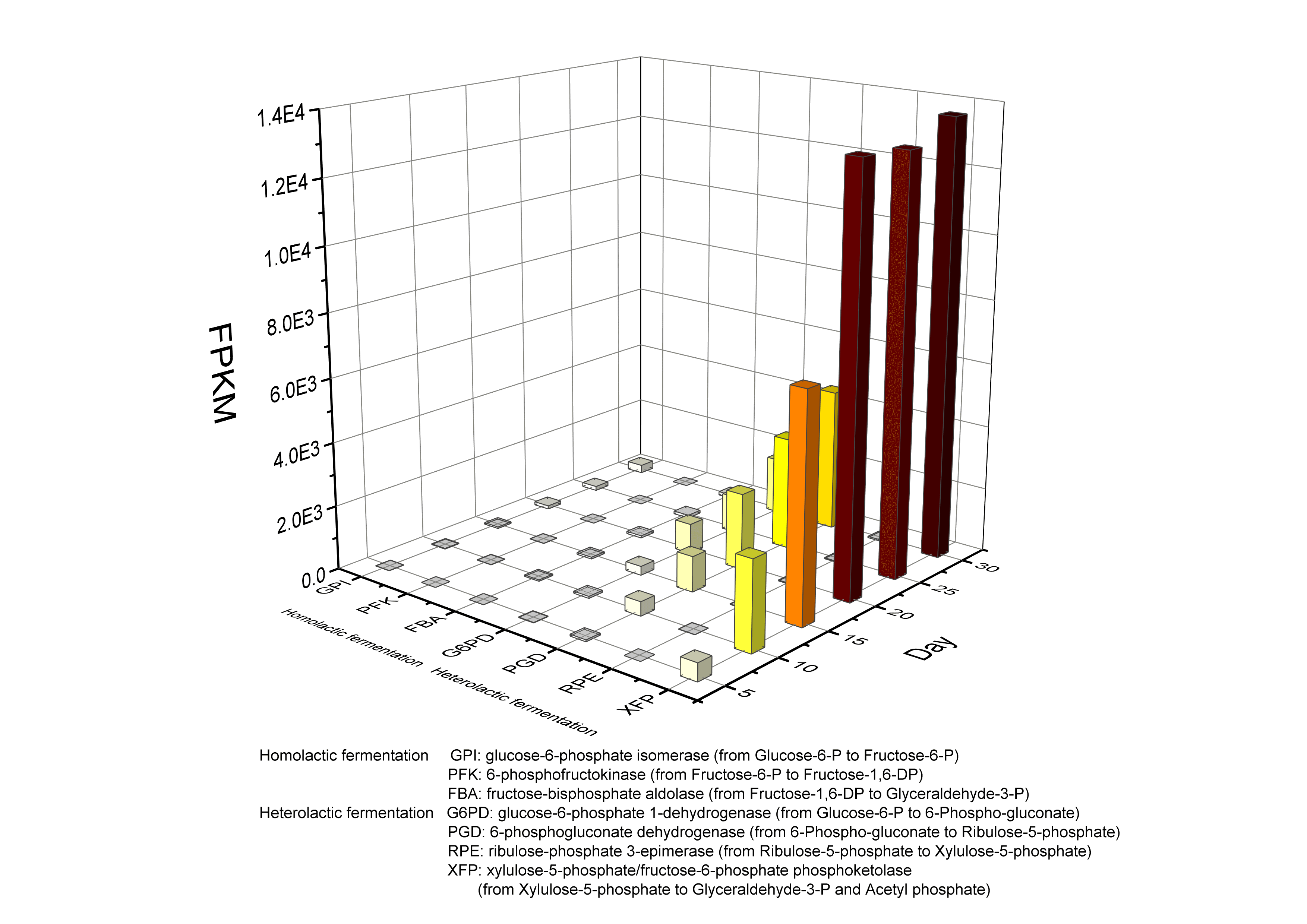


**Figure S7.** The gene expression of key enzymes in lactic acid metabolism about the genus *Lactobacillus* (n = 6).

**
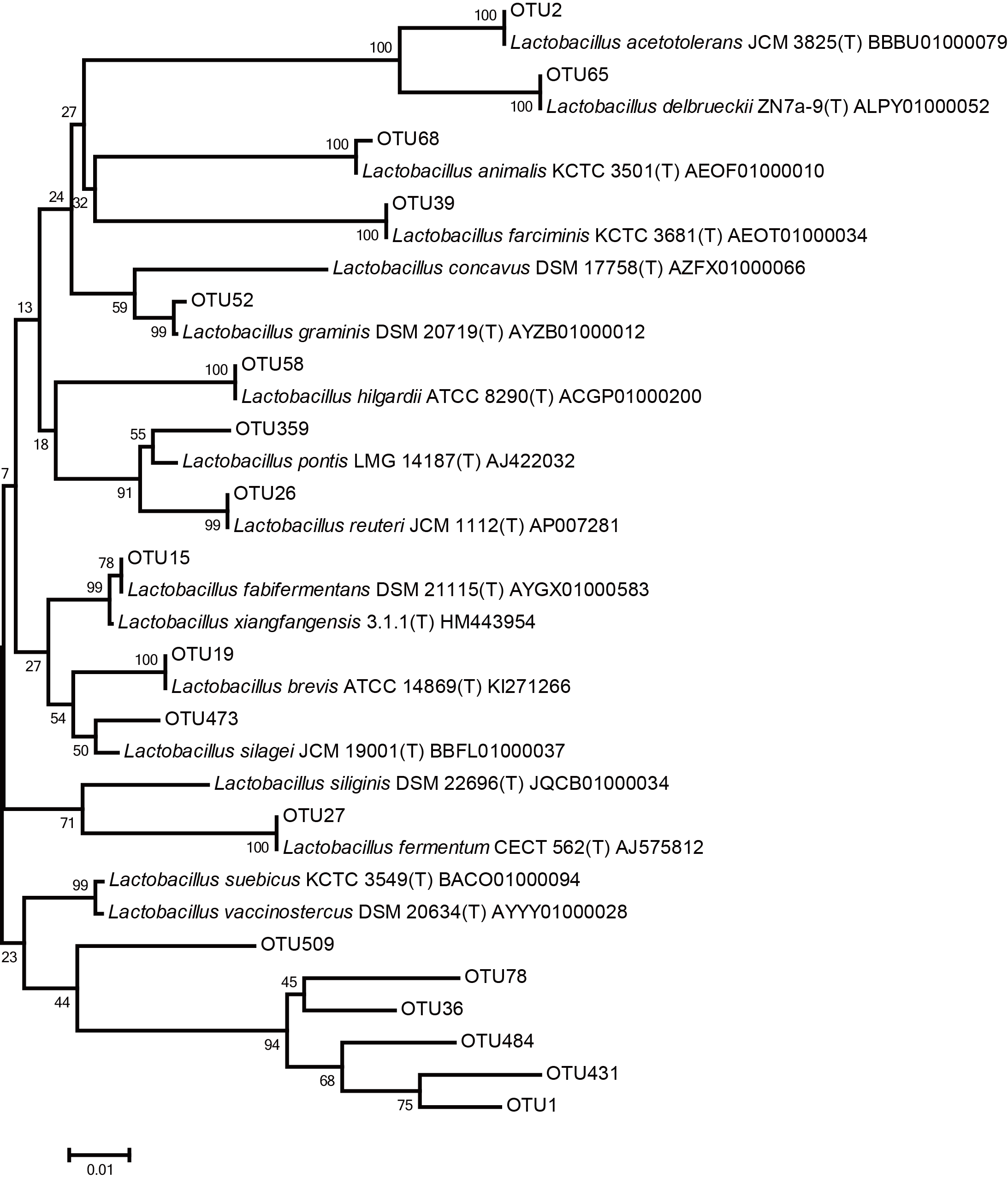
Figure S8.** Phylogenetic tree of lactobacilli (Taxa>0.02%) based 16S rRNA amplicons database in Chinese soy sauce liquor production.


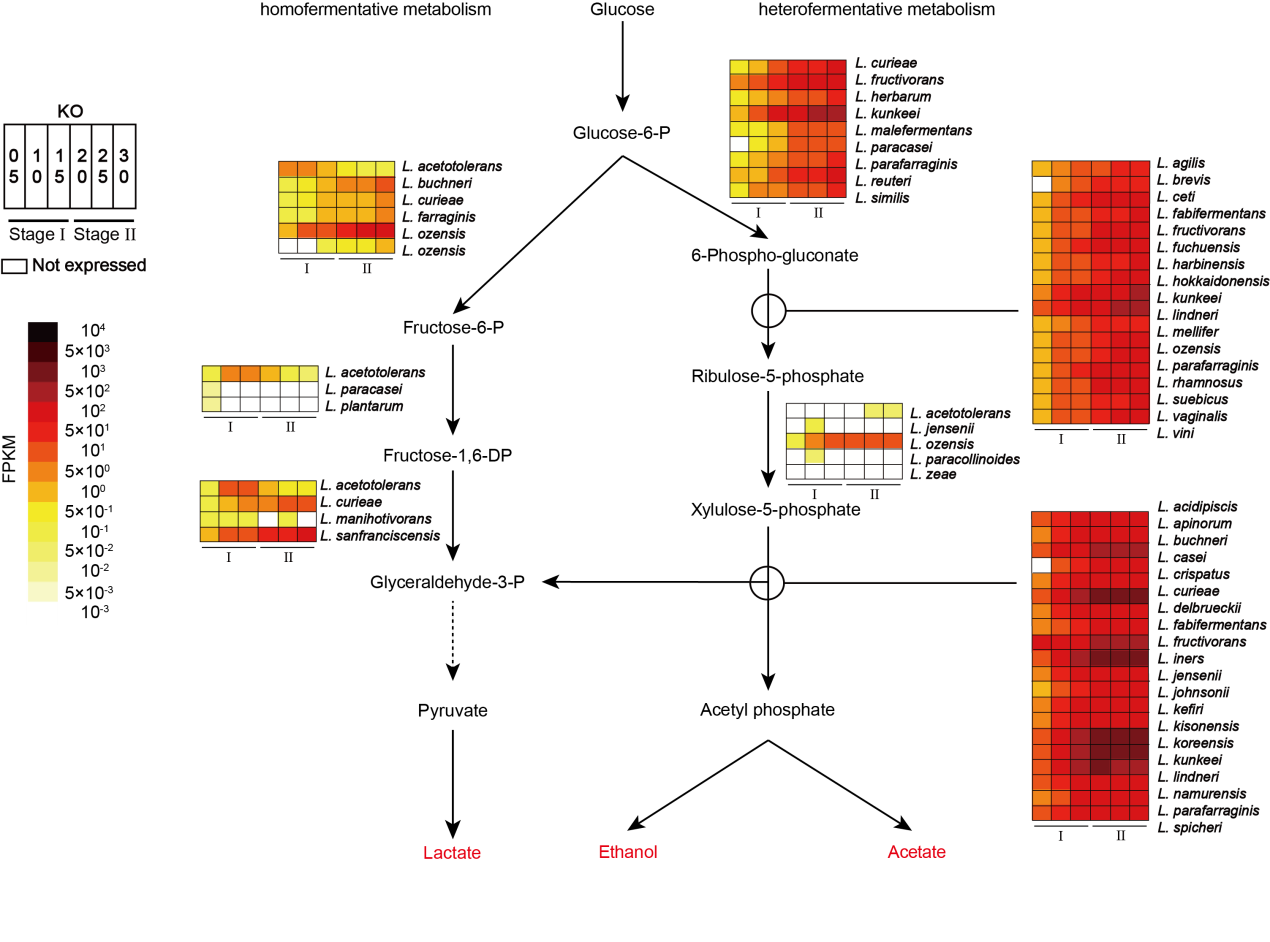


Figure S9. The gene expression of homofermentative and heterofermentative metabolism related to lactobacilli in Chinese soy sauce liquor production (n = 6).

- 1. **Supplementary Tables**

**Table S1.** Summary of diversity index for bacterial microbiota based on 16S rRNA amplicons sequencing across samples.

| Samples (n = 18) | Day 5 (n = 3) | Day 10 (n = 3) | Day 15 (n = 3) | Day 20 (n = 3) | Day 25 (n = 3) | Day 30 (n = 3) |
| --- | --- | --- | --- | --- | --- | --- |
| Clean reads | 15,638 ± 2,386 | 16,615 ± 1,350 | 33,400 ± 10,466 | 42,532 ± 19,414 | 41,830 ± 4,163 | 90,160 ± 19,594 |
| OTU number | 278 ± 52 | 280±55 | 289 ± 22 | 274 ± 46 | 216±23 | 236 ± 17 |
| Goods coverage (%) | 99.55 ± 0.07 | 99.54 ± 0.02 | 99.55 ± 0.02 | 99.63 ± 0.10 | 99.73 ± 0.05 | 99.74 ± 0.02 |
| Chao1^a^ | 270.07 ± 38.51 | 296.22 ± 23.22 | 281.61 ± 30.30 | 240.66 ± 58.11 | 191.17 ± 24.73 | 160.07 ± 3.03 |
| Shannon^a^ | 4.54 ± 0.69 | 2.76 ± 1.78 | 1.44 ± 0.82 | 1.26 ± 1.15 | 0.42 ± 0.16 | 0.26 ± 0.04 |

^a^ Except for the number of good quality sequences, the other indices were calculated based on cut-off = 0.03 and 12426 sequences per sample.

**Table S2.** Summary of diversity index for fungal microbiota based on ITS amplicons sequencing across samples.

| Samples (n = 18) | Day 5 (n = 3) | Day 10 (n = 3) | Day 15 (n = 3) | Day 20 (n = 3) | Day 25 (n = 3) | Day 30 (n = 3) |
| --- | --- | --- | --- | --- | --- | --- |
| Clean reads | 53,118 ± 22,012 | 35,145 ± 10,150 | 56,870 ± 10,326 | 59,469 ± 16,070 | 56,681 ± 18,929 | 42,981 ± 2,248 |
| OTU number | 140 ± 37 | 164 ± 53 | 209 ± 20 | 212 ± 21 | 186 ± 49 | 247 ± 28 |
| Goods coverage (%) | 99.89 ± 0.02 | 99.83 ± 0.06 | 99.87 ± 0.01 | 99.84 ± 0.04 | 99.84 ± 0.04 | 99.82 ± 0.02 |
| *Chao1*^a^ | 153.91 ± 37.31 | 194.71 ± 72.14 | 224.11 ± 19.76 | 256.36 ± 24.01 | 210.42 ± 44.40 | 262.16 ± 4.60 |
| *Shannon*^a^ | 1.77 ± 0.12 | 2.31 ± 0.36 | 2.10 ± 0.09 | 2.39 ± 0.33 | 1.95 ± 0.22 | 3.09 ± 0.55 |

^a^ Except for the number of good quality sequences, the other indices were calculated based on cut-off = 0.03 and 27470 sequences per sample.
